# Gastrointestinal Shedding of Rubulaviruses from Egyptian Rousette Bats: Temporal Dynamics and Spillover Implications

**DOI:** 10.3390/microorganisms12122505

**Published:** 2024-12-04

**Authors:** Tauya S. Muvengi, Marinda Mortlock, Morgan P. Kain, Wanda Markotter

**Affiliations:** 1 Centre for Viral Zoonoses, Department of Medical Virology, School of Medicine, Faculty of Health Sciences, University of Pretoria, Pretoria 0001, South Africamarinda.mortlock@up.ac.za (M.M.); 2 Independent Researcher, Washington, DC 20002, USA

**Keywords:** rubulaviruses, Egyptian rousette bats, South Africa, human parainfluenza virus 2, viral dynamics

## Abstract

Bats are recognized as reservoirs for diverse paramyxoviruses, some of which are closely related to known human pathogens or directly implicated in zoonotic transmission. The emergence of the zoonotic Sosuga virus (SOSV) from Egyptian rousette bats (ERBs), which caused an acute febrile illness in a reported human case in Africa, has increased the focus on the zoonotic potential of the *Rubulavirinae* subfamily. Previous studies identified human parainfluenza virus 2 (HPIV2)- and mumps (MuV)-related viruses in ERBs from South Africa, with HPIV2-related viruses restricted to gastrointestinal samples, an underexplored target for rubulavirus biosurveillance, suggesting that sample-type bias may have led to their oversight. To address this, we performed a longitudinal analysis of population-level fecal samples from an ERB maternity roost for rubulavirus RNA, employing a broadly reactive hemi-nested RT-PCR assay targeting the polymerase gene. We detected HPIV2- and MuV-related viruses in addition to numerous pararubulaviruses, highlighting significant viral diversity. Temporal analysis of three major clades revealed peaks in rubulavirus shedding that correlated with seasonal environmental changes and host reproductive cycles, although shedding patterns varied between clades. These findings identify specific periods of increased risk for the spillover of bat-associated rubulaviruses to humans, providing critical information for developing targeted mitigation strategies to minimize zoonotic transmission risk within the local community.

## 1. Introduction

Bats are recognized as natural reservoirs for a number of paramyxoviruses, including notable zoonotic pathogens such as Hendra and Nipah viruses belonging to the *Henipavirus* genus in the *Orthoparamyxovirinae* subfamily [1,2,3]. Although rubulaviruses were the first of the paramyxoviruses to be associated with bats, rubulavirus surveillance has remained significantly less extensive compared to the orthoparamyxoviruses [4,5,6,7,8,9,10,11,12]. Nonetheless, several rubulaviruses have been detected from different bat species, predominantly fruit bats, from various localities across the globe. These include the Tioman virus from Malaysia [6], the Tuhoko virus from China [7], and the Achimota virus from Ghana [9,10]. As these viruses were being fully classified, the genus *Rubulavirus* was elevated to the subfamily level (*Rubulavirinae*) and two new genera, i.e., *Ortho*- and *Pararubulavirus*, were established to accommodate the increasing number of viruses [3].

Rubulaviruses have been associated with several significant human and animal diseases including the major human pathogens mumps (MuV), human parainfluenza virus 2 (HPIV2; *Orthorubulavirus*) and Menangle virus (*Pararubulavirus*), a bat-associated virus causing devastating disease in pigs and suspected human cases of a flu-like disease [5,13,14,15]. Sosuga virus (SOSV) (*Pararubulavirus*) is the only rubulavirus directly associated with a zoonotic disease in humans, with the Egyptian rousette bat (ERB; *Rousettus aegyptiacus*) implicated as the natural reservoir [16,17,18,19]. Egyptian rousette bats have since been identified as natural hosts for a range of rubulaviruses, some of which are related to the human pathogens in the *Orthorubulavirus* genus mentioned previously [5,11,12,18,19].

Mortlock et al. (2019) detected diverse rubulaviruses from a longitudinal urine excretion study supplemented with tissue distribution analysis and performed in ERBs from a South African population. Two major peaks of active virus excretion were identified, which coincided with the host reproduction cycle, though viral excretion was only assessed collectively across the *Rubulavirinae* subfamily [11]. Viruses from both rubulavirus genera were described, including MuV- and HPIV2-related viruses, the latter exclusively detected in a small number of gastrointestinal samples tested during the tissue distribution analysis. This highlights the importance of including gastrointestinal samples in rubulavirus biosurveillance studies to comprehensively characterize the viral diversity associated with this bat species.

To expand on the hypothesis that the gastrointestinal route can serve as a prominent pathway of excretion for HPIV2-related rubulaviruses, this study aimed to examine the excretion dynamics of rubulaviruses in longitudinally collected fecal samples from the same ERB population in South Africa previously studied by Mortlock et al. [11]. Additionally, this study sought to determine whether peaks in viral excretion are driven by specific viral groups, thereby contributing to a more detailed understanding of rubulavirus dynamics in ERBs.

## 2. Materials and Methods

### 2.1. Study Site, Ethical Clearances and Biosafety Considerations

**Figure 1:**
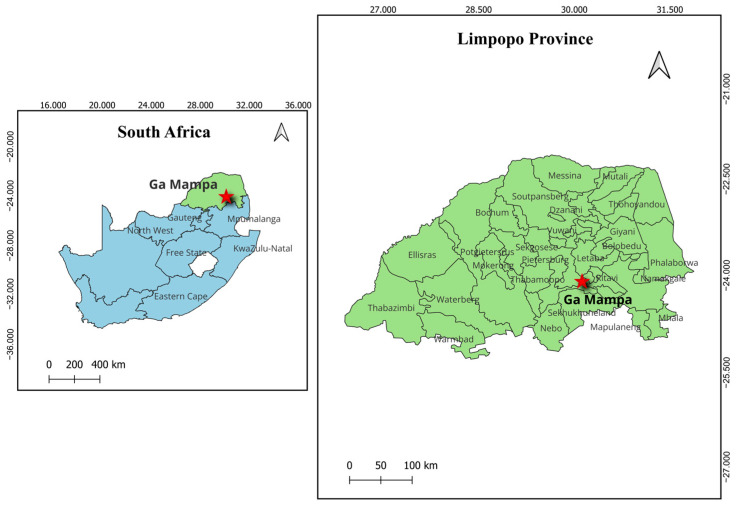
Map showing the location of the sampling area in Limpopo province, South Africa. The location of Ga Mampa is marked with a red asterisk.

The targeted site for sampling ERBs in South Africa was the same as previously described [11]. Briefly, ERBs roosted in the Matlapitsi cave, Ga Mampa, Limpopo Province, South Africa, situated in a rural community with free-roaming domestic animals, livestock and other wildlife (Figure 1).

Authorization to conduct disease research on animals was granted by the Department of Agriculture, Land Reform and Rural Development (DALRRD) of South Africa under Section 20 of the Animal Disease Act (Act No. 35 of 1984). Moreover, Limpopo sampling permits (CPM 06806; ZA/LP/84188 and ZA/LP/91509; ZA/LP/100499) from the Department of Economic Development, Environment and Tourism of the Limpopo Provincial Government and ethical clearances (552/2020; EC054-14; 145/2021) from the University of Pretoria Animal Ethics committee were obtained. All samples were collected under strict biosafety conditions using the appropriate personal protective equipment (PPE) such as Tyvek coveralls (DuPont, Richmond, VA, USA), gumboots, powered air-purifying respirators (PAPRs) (3M, St. Paul, MN, USA) and double-layer nitrile gloves. After sampling, all equipment and clothing were decontaminated using a 10% bleach solution.

### 2.2. Sample Collection and Viral Screening

Pooled population-level fecal samples (*n* = 1013) were collected as environmental samples from the cave floor underneath roosting ERBs using large, cotton-tipped, sterile swabs (VWR Critical Swab, Radnor, PA, USA). Three fecal boluses were smeared off into 2 mL microcentrifuge tubes (SARSTEDT, ThermoFisher Scientific, Waltham, MA, USA) with 1X DNA/RNA shield (Zymoresearch, Irvine, CA, USA) to represent one sample pool. All samples were immediately frozen in a liquid-nitrogen-charged cryo-shipper (MVE Biological Solutions LLC, Ball Ground, GA, USA) and subsequently stored in a −80 °C freezer (NuAire Laboratory Equipment, Plymouth, MN, USA) after transport to the laboratory. Retrospective samples collected monthly from June 2017 to December 2019 were selected for testing (representing 31 consecutive months). A detailed sample list is provided in Table S1.

A subset of pooled fecal samples was previously extracted using the Quick-RNA™ MiniPrep Plus kit (Zymoresearch, Irvine, CA, USA) as part of another study [20]. The remainder of the samples were extracted in a BSL-3 facility using the NucleoMag^®^ VET RNA/DNA extraction kit (Macherey-Nagel, Düren, Germany) following the manufacturer’s recommendations. Nucleic acid amplification was performed using the *Avula*- and *Rubulavirinae* (AR) assay, as previously described [11]. This broadly reactive primer set targets the partial polymerase (L) gene of the *Avula*- and *Rubulavirinae* subfamilies and has previously been used with success in rubulavirus surveillance [5,11].

### 2.3. Bioinformatic Analyses

For phylogenetic analysis, the nucleotide alignment was run in the jModel-Test software (v2.1.10 Universidade de Vigo, Vigo, Spain) to infer the best DNA substitution model. Subsequently, Bayesian phylogenetic analysis was performed in BEAST (v2.5.1, Beast 2 development team 2011–2018) using the recommended best-fit model with 10,000,000 iterations and sampling every 1000 trees [21]. The tree was visualized in TreeAnnotator v2.5.1 (within the BEAST package; v2.5.1, Beast 2 development team 2011–2018) with a burn-in value of 10% and labeled using FigTree v1.4.2 (2006–2012 Andrew Rambaut, Institute of Evolutionary Biology, University of Edinburgh) [22]. To assess similarities at nucleotide and amino acid levels, identity plots were generated using the BioEdit sequence alignment editor (v7.2.5) [23]. Additionally, sequences were analyzed using the Basic Local Alignment Search Tool (BLAST v2.16.0) against the NCBI nucleotide database (accessible online at https://blast.ncbi.nlm.nih.gov/Blast.cgi accessed on 16 July 2024) to identify homologous sequences and determine the closest known relatives. A BLAST search was conducted with default parameters to ensure comprehensive comparison and accurate taxonomic identification.

### 2.4. Temporal Excretion Analysis

A Generalized Additive Model (GAM) with a binomial error and a logit link function was used to assess the longitudinal excretion dynamics of three larger clusters within the *Rubulavirinae* subfamily. We used two smoothing terms to characterize temporally variable excretion dynamics: (1) a cyclic cubic regression spline (to constrain the start and end of the smooth to align) to capture within-year seasonal excretion patterns and (2) a thin plate regression spline for long-term changes in virus prevalence across the full duration of the study. Term one was fit using the Julian date and term two was fit with the cumulative days since the start of sampling. We used an interaction term between both smooths and the major phylogenetic clades to model variable excretion patterns by phylogenetic clade. All analysis was performed in R version 4.3.0 [24].

## 3. Results

### 3.1. Rubulavirus Positivity, Sequence Identity and Phylogeny

Rubulavirus RNA was detected in 5.8% (*n* = 59) of the tested 1013 population-level fecal pools. Although the samples were pooled, a single rubulavirus sequence was detected per sample pool, with no co-detections observed. Detailed information on all tested samples, results and Genbank accession numbers can be found in Table S1. Upon BLAST analyses, the detected virus sequences were closely related to MuV (*n* = 5) and HPIV2 (*n* = 35) from the *Orthorubulavirus* genus, while the rest were all within the *Pararubulavirus* genus (*n* = 19) (Table 1). The latter was grouped as one cluster as the diversity in this genus is quite substantial and many of the described viruses have not been characterized. The viruses detected in this cluster were not specifically closely related to any of the described pararubulaviruses. After sequences sharing 100% sequence identity were grouped together, 21 unique viral sequences were identified in this study. Comparative similarity analysis of the 21 unique sequences indicated a nucleotide identity ranging between 70 and 80% and an amino acid identity of 76–97% with characterized rubulavirus species (Table 1). Identities of 90% and higher at the amino acid level (structural/functional level) were observed for sequences related to MuV and HPIV2. No RNA of the zoonotic SOSV was detected in the population, with the closest sequences sharing identities of 79% and 82% with SOSV on the amino acid level.

In a comparative analysis with the rubulavirus sequences previously reported for the same population of ERBs at the Matlapitsi cave [11], nucleotide identities ranged from 61 to 99%, with only one sequence (UPE1839_R_aeg_fecal_pooled(4)) sharing 100% identity with previously reported sequences (Table S2). However, when considering amino acid identities, an additional five sequences shared 100% identity. In total, 15 novel rubulavirus sequences were described from ERB fecal samples in this study. For the MuV-related diversity, both an identical sequence and an additional variant sharing 98% amino acid identity to what was previously described for the ERB population were detected. At least eight additional viral sequence variants related to HPIV2 were additionally detected in this study (sharing less than 99% amino acid identity to the previously reported HPIV2-related virus from the same population).

**Figure 2:**
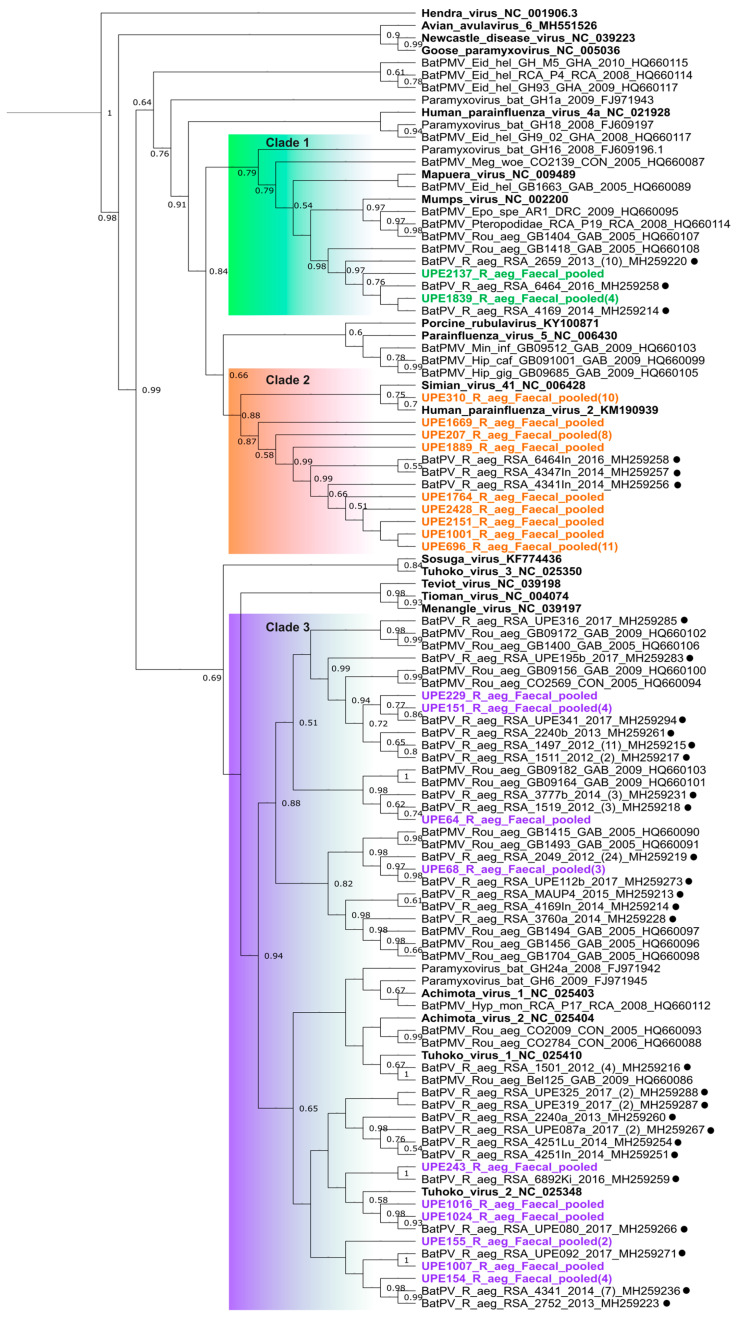
A Bayesian phylogeny constructed using the partial polymerase gene sequences (186 nucleotides) of the rubulaviruses detected in Egyptian rousette bat fecal samples using an *Avula-Rubulavirinae* specific assay. The phylogeny was constructed in BEAST v2.5.1. using the transversion model with a gamma distribution and invariant sites (TVM + I + G). The phylogenetic tree was captured in proportional view, and posterior probabilities > 0.5 are shown at internal nodes. Colored sequences were detected in this study. Sequences from the same bat population from a previous study are indicated with a dot. Sequences from characterized viral species are indicated in boldface. The numbers in brackets at the end of each sequence represent the number of detections per sequence. Rubulavirus clades are highlighted in green (mumps-related viruses), orange (human parainfluenza 2-related viruses) and purple (genus *Pararubulavirus*-related viruses).

Phylogenetic analyses of sequences detected in this study indicated three genetically distinct clades representing the MuV- and HPIV2-related viruses in the *Orthorubulavirus* genus and one representing the genus *Pararubulavirus* (Figure 2, clades 1 to 3, respectively).

Sufficient phylogenetic support was obtained at internal branching points separating the two rubulavirus genera *Ortho*- and *Pararubulavirus* (posterior probability of 0.99). As such, analyses indicated with certainty that the viral sequences detected in this study were grouped under both the *Ortho*- and *Pararubulavirus* genera (Figure 2). The MuV-related sequences clustered with other sequences that were detected in ERBs in South Africa and Gabon, seemingly forming a genus-specific sub-clade (Figure 2, clade 1). The majority of sequences in the *Orthorubulavirus* genus were grouped with HPIV2, Simian virus 41 (SV41) (Figure 2, clade 2) and other sequences that were detected from intestine samples from a previous study on the same ERB population (amino acid identity > 90%) [11]. One virus sequence, UPE310_R_aeg_fecal_pooled(10), in the HPIV2-related clade, was most similar to HPIV2 (amino acid identity 95%) (Table 1; Figure 2, clade 2) compared to the other bat-borne HPIV2-related virus sequences reported here.

The low phylogenetic resolution (posterior values < 0.5) observed within the *Pararubulavirus* genus limited inferences regarding the phylogenetic placement of the detected sequences relative to all included classified viral species (Figure 2, clade 3). A large proportion of sequences phylogenetically were grouped with others detected from urine and organ samples from the same ERB population as well as those from the same species sampled in different regions in Africa [5,11]. No geographic clustering was observed between sequences detected from the South African colony and others from distant locations on the African continent.

### 3.2. Temporal Analysis of Rubulavirus Excretion

**Figure 3:**
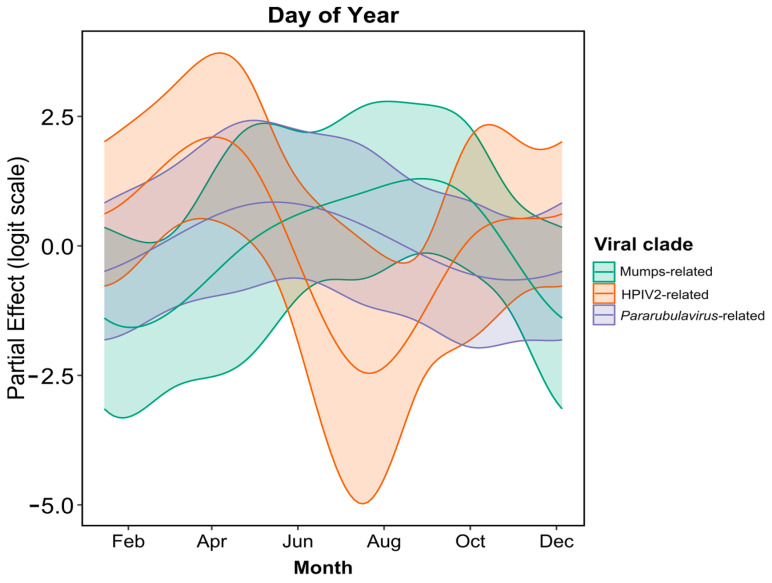
Predicted within-year variation among three rubulavirus clades detected in Egyptian rousette bat fecal sample pools.

A temporal assessment of rubulavirus excretion was performed to look at within-year variation within the three identified clades, i.e., MuV- and HPIV2- and *Pararubulavirus* genus-related (Figure 2, clades 1 to 3). The analysis indicated significant within-year variation in all three groups (Table 2). Additionally, after accounting for within-year seasonal variation, additional significant variation was detected across the study period for the pararubulavirus clade; no significant long-term temporal trends (apart from seasonality) were observed for the MuV-related and HPIV2 viral clades (Table 2, Figure 3).

**Figure 4:**
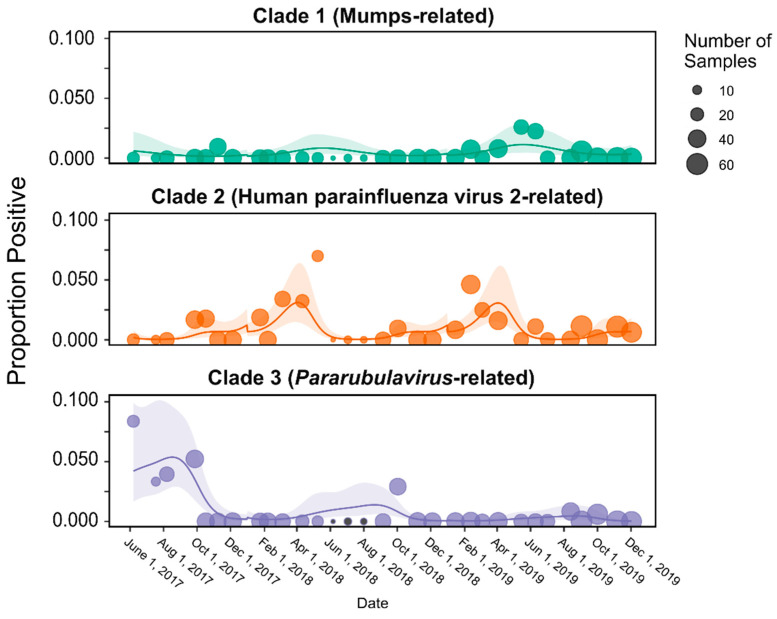
Predicted monthly prevalence of virus sequences in the three rubulavirus clades detected over a period of 2.5 years. Dots represent the proportion of positive samples and shaded areas represent 95% confidence intervals. Different colors represent the different clades and correspond to the same-colored clades depicted in Figure 2.

Periods of increased detection of HPIV2-related viruses were observed between February and May, i.e., the autumn season for the region. The excretion dynamics for the pararubulaviruses were different from what was observed for HPIV2-related viruses, as peaks in viral excretion and RNA detection were observed from June to August, corresponding to the winter season (Figure 4). Despite a significant smoothing term for MuV-related sequences (Table 2), rejecting the hypothesis of constant shedding year-round, the low number of detections of MuV-related viral sequences made it difficult to pinpoint precisely when peak excretion occurred (Figure 3).

## 4. Discussion

With the rise of biosurveillance studies on wildlife species such as bats, the selection of target sample types has largely been exploratory or based on knowledge of specific pathogens in other host species. Consequently, studies have often focused on particular sample types, such as gastrointestinal samples for coronaviruses and brain or oral swabs for lyssavirus surveillance [20,25,26,27]. As more research emerges, alternative approaches are being explored, such as using fecal material for bat lyssavirus surveillance, which is considered a more practical and feasible option [28]. A similar trend is evident in paramyxovirus surveillance, where urine samples have proven suitable across viral genera and host species and have thus been the focus of most research studies [8,9,10,11]. However, previous rubulavirus research in ERBs has indicated that this sample type bias may hinder the detection of certain rubulavirus species, such as HPIV2-related viruses [11]. In this study, by employing a longitudinal collection of fecal material from beneath roosting ERBs, we confirmed the presence of diverse HPIV2-related rubulaviruses in these samples, supporting the hypothesis of a gastrointestinal association for these viruses in ERBs. Given these findings, along with prior reports of SOSV excretion in gastrointestinal samples, this evidence highlights the importance of considering this potential transmission route in future rubulavirus investigations [18,19].

The excretion of viruses from bats through feces presents a significant risk of human transmission, as direct contact with bats is not necessary for exposure; instead, it is more likely to occur indirectly through environmental contamination [18]. Thanks to the availability of longitudinal fecal samples, this study was able to provide preliminary data on the temporal excretion patterns of specific rubulavirus clades, identifying periods of heightened risk for the local population. A significant seasonal variation was observed across all three phylogenetic clusters, though only the *Pararubulavirus*-related cluster showed notable variation over the entire study period, with higher positivity at the beginning and a decline in later stages. The risk of exposure to pararubulaviruses and MuV-related viruses was highest during winter, aligning with previous rubulavirus data from urine samples collected from the same ERB population [11]. Winter in this region is characterized by cold temperatures and minimal rainfall, which limits natural food resources and subjects the bats to physiological and nutritional stress [11,29]. This stress could weaken their immune systems, making them more susceptible to infection and increasing viral shedding [11,20,30,31]. Seasonal tracking data from ERBs in Ga Mampa reported that the bats frequent residential areas during the dry winter months to forage on cultivated fruit trees, increasing the likelihood of contact with humans and domesticated animals and thereby elevating the risk of zoonotic spillover [32]. In contrast, HPIV2-related virus sequences were detected throughout most months, with a peak in autumn, coinciding with the weaning period of ERB pups [29,33]. However, no detections of HPIV2-related virus sequences were observed during winter months, potentially suggesting viral competition [34,35]. The peak in HPIV2-related virus excretion also coincides with the presumed decline in maternal immunity in pups over six months of age, making them more susceptible to viral infection and transmission [36], a pattern previously reported for the Marburg virus in the same bat population in Ga Mampa [31]. Interestingly, Geldenhuys et al. [20] also reported an autumn peak in beta- and alphacoronavirus excretion, with a similar gastrointestinal association, using the same population-level fecal samples, of which nine pools were dually positive for both corona- and rubulaviruses. Intermittent excretion of HPIV2-related viruses observed during the spring and summer months suggests the ongoing maintenance of these viruses within the bat population.

It is evident from this and numerous other studies that viral activity fluctuates seasonally within bat populations [11,20,30]. While cross-sectional studies provide value in certain research contexts, they have significant limitations when applied to the biosurveillance of viruses in bats. For example, if the timing of the study does not align with periods of peak virus excretion, circulating viruses may go undetected, or viral diversity may be underestimated. For resource-limited countries, the cross-sectional approach remains the most affordable option to initiate biosurveillance efforts. However, the success of rubulavirus biosurveillance through off-host urine and fecal samples demonstrates that capturing and handling animals—along with the stringent ethical requirements and significant human resource demands this entails—can be avoided. Given the advantages of off-host sampling, its application in longitudinal research offers a more attractive and feasible option for many resource-limited countries, providing improved data on viral dynamics without the need for invasive techniques. However, additional research, such as full-genome sequencing and virus characterization, may be hindered by the limited size of collected sample material in conjunction with the low viral titers, especially for smaller host species.

Although the zoonotic potential of the bat-associated rubulaviruses described remains unknown, the detection of viral sequences with high genetic similarity to known human pathogens, such as mumps virus (MuV) and human parainfluenza virus 2 (HPIV2), raises concerns. An experimental study using a recombinant pseudoparticle derived from a bat-associated MuV (batMuV), previously detected in an Epauletted fruit bat (*Epomophorus* sp.) in the Democratic Republic of Congo [5], demonstrated that batMuV can actively infect and replicate in human cells [37]. The cross-neutralization of recombinant batMuV by anti-human MuV antibodies suggests that existing vaccination against human MuV may provide some protection against batMuV. However, with the decline in childhood vaccinations following the COVID-19 pandemic [38] and the resulting decrease in population immunity to viruses like MuV, there may be increased human susceptibility to bat-borne counterparts. Given the high genetic similarity between HPIV2 and the bat-associated strains, it is plausible that these viruses could also infect and replicate in human cells, though this hypothesis requires support through experimental studies. The recent identification of a complete genome of an HPIV2 virus associated with ERBs [39] offers a unique opportunity. By applying recombinant pseudoparticles to assess the infectivity and replication of this bat-derived HPIV2 strain in human cells, future studies can help elucidate its potential zoonotic risk.

The detection of highly similar viruses in ERBs across their sub-Saharan African range and the observed phylogenetic clustering of sequences from different countries, such as the Democratic Republic of Congo and Gabon [5], suggest a long history of co-evolution between ERBs and rubulaviruses [40]. Consequently, the findings from this study are relevant beyond the local community of Ga Mampa. However, the drivers behind the observed rubulavirus peaks in the South African ERB population remain unclear. Variations in environmental conditions and differences in host reproductive biology across the ERB distribution range [28,36,41] could lead to divergent seasonal dynamics. As a result, periods of increased risk of exposure may not be consistent across different regions. Nonetheless, this study highlights the importance and need for continued rubulavirus surveillance in bats, with particular reference to ERBs.

## 5. Conclusions

The exclusive detection of HPIV2-related viruses in gastrointestinal samples across two longitudinal studies of the targeted ERB population underscores the limitations associated with sample type bias. To address this, we recommend that the design of rubulavirus biosurveillance studies incorporates the longitudinal collection and testing of both off-host urine and fecal samples to enhance coverage and capture the full spectrum of rubulavirus diversity. Furthermore, transdisciplinary research focused on understanding rubulavirus excretion dynamics in ERBs across their geographical range, as well as the impact of climatic and environmental changes on host–pathogen interactions, is crucial. Such studies will help identify key drivers of viral excretion, thereby contributing to evidence-based regional risk assessments and the development of targeted mitigation strategies.

## Figures and Tables

**Table 1:** Virus sequences detected and partial polymerase gene sequence nucleotide and amino acid identities shared with officially characterized *Rubulavirinae* species.

Representative Virus Sequence (Number of Detections)	Additional Positive Samples *	Highest Similarity (%) to Classified Rubulavirus Species ^#^			
		Virus	Nucleotide	Virus	Amino Acid
UPE64_R_aeg_fecal_pooled	-	ThkPV1	73%	AchPV2	85%
UPE68_R_aeg_fecal_pooled (3)	UPE071; UPE074	ThkPV1/AchPV2	73%	AchPV2	82%
UPE151_R_aeg_fecal_pooled (4)	UPE212; UPE224; UPE236	AchPV2	80%	ThkPV2/AchPV1	82%
UPE154_R_aeg_fecal_pooled (4)	UPE214; UPE2112; UPE2340	ThkPV1/AchPV2	70%	ThkPV2	82%
UPE155_R_aeg_fecal_pooled (2)	UPE110	AchPV2/HPIV4a	73%	AchPV1	76%
UPE207_R_aeg_fecal_pooled (8)	UPE210; UPE294; UPE1483; UPE1551; UPE1556; UPE1558; UPE1667	HPIV2	80%	**HPIV2**	**90%**
UPE229_R_aeg_fecal_pooled	-	AchPV2	75%	ThkPV1/2	81%
UPE243_R_aeg_fecal_pooled	-	ThkPV2/SOSV	73%	SOSV	79%
UPE310_R_aeg_fecal_pooled (10)	UPE386; UPE776; UPE780; UPE781; UPE1568; UPE1570; UPE1582; UPE1586; UPE1745	HPIV2	75%	**HPIV2**	**95%**
UPE696_R_aeg_fecal_pooled (11)	UPE541; UPE697; UPE698; UPE750; UPE751; UPE1749; UPE1875; UPE2116; UPE2446; UPE2585	HPIV2/SV41	78%	HPIV2	94%
UPE1001_R_aeg_fecal_pooled	-	HPIV2	78%	**HPIV2**	**92%**
UPE1007_R_aeg_fecal_pooled	-	HPIV4a	77%	SOSV	82%
UPE1016_R_aeg_fecal_pooled	-	AchPV1	76%	ThkPV2	82%
UPE1024_R_aeg_fecal_pooled	-	ThkPV2	76%	AchPV1	82%
UPE1669_R_aeg_fecal_pooled	-	PIV5	76%	HPIV2	87%
UPE1764_R_aeg_fecal_pooled	-	HPIV2	74%	**HPIV2**	**90%**
UPE1839_R_aeg_fecal_pooled (4)	UPE563; UPE1845; UPE1872	MuV	76%	**MuV**	**97%**
UPE1889_R_aeg_fecal_pooled	-	HPIV2	76%	**HPIV2**	**87%**
UPE2137_R_aeg_fecal_pooled	-	MuV	75%	**MuV**	**95%**
UPE2151_R_aeg_fecal_pooled	-	HPIV2/SV41	79%	**HPIV2**	**92%**
UPE2428_R_aeg_fecal_pooled	-	HPIV2	77%	**HPIV2**	**90%**

* Sequences grouped together shared a 100% nucleotide identity. ^#^ Species abbreviations: AchPV1—Achimota virus 1 (NC_025404.1); AchPV2—Achimota virus 2 (NC_025404); HPIV2—Human parainfluenza 2 (KM190939); MuV—Mumps virus (NC_002200); SV41—Simian virus 41 (NC_006428); SOSV—Sosuga virus (KF774436.1); ThkPV1—Tuhoko virus 1 (NC_025410); ThkPV2—Tuhoko virus 2 (NC_025348). Boldface entries represent instances where amino acid identities were 90% or more.

**Table 2:** Temporal excretion patterns of different rubulavirus clades across the sampling period.

Viral Cluster Analyzed	Chi-Square	*p*-Value ^#^
*Within-year variation*		
Mumps-related	5.243	0.026279 *
Human parainfluenza 2-related	29.108	3.26 × 10^−7^ ***
*Pararubulavirus*-related	14.894	3.72 × 10^−4^ ***
*Variation across the study period*		
Mumps-related	0.616	0.432806
Human parainfluenza 2-related	0.002	0.969013
*Pararubulavirus*-related	21.155	3.01 × 10^−5^ ***

^#^ Significance level: * significant < > *** highly significant.

## Data Availability

The original data presented in this study are provided in the Supplementary Materials associated with the publication. All sequences used for data and phylogenetic analyses are deposited in the National Center for Biotechnology Information (NCBI) Genbank database (available online at https://www.ncbi.nlm.nih.gov/genbank/ accessed on 9 October 2024). Genbank accession numbers for the sequences identified in this study are MW118270 to MW118278, OR365870 to OR365873, OR365875, and OR365877 to OR365920. The code used for analyses is added as the Supplementary file Rubulavirus_RScript1.

## Supplementary Materials

The following supporting information can be downloaded at https://www.mdpi.com/article/10.3390/microorganisms12122505/s1: Table S1: Sample information; Table S2: Sequence identities.
