# Supplementary material for: Gastrointestinal Shedding of Rubulaviruses from Egyptian Rousette Bats: Temporal Dynamics and Spillover Implications
Source: Microorganisms. 2024 Dec 4;12(12):2505. doi: 10.3390/microorganisms12122505 (PMC11728649; doi:10.3390/microorganisms12122505)
Supplement: Supplementary file 1 [file microorganisms-12-02505-s001.zip › Muvengi et al. 2024_Table S1_Sample information.pdf]

Table S1: Egyptian rousette bat population-level faecal samples tested for rubulavirus RNA

Laboratory number	Source/Host	Sample type	Date collected	Result	Genbank Accession Number
UPE 061	*Rousettus aegyptiacus*	Population-level faecal	7-Jun-17	Negative	
UPE 062	*Rousettus aegyptiacus*	Population-level faecal	7-Jun-17	Negative	
UPE 063	*Rousettus aegyptiacus*	Population-level faecal	7-Jun-17	Negative	
**UPE 064**	***Rousettus aegyptiacus***	**Population-level faecal**	**7-Jun-17**	**Positive**	**MW118275**
UPE 065	*Rousettus aegyptiacus*	Population-level faecal	7-Jun-17	Negative	
UPE 066	*Rousettus aegyptiacus*	Population-level faecal	7-Jun-17	Negative	
UPE 067	*Rousettus aegyptiacus*	Population-level faecal	7-Jun-17	Negative	
**UPE 068**	***Rousettus aegyptiacus***	**Population-level faecal**	**7-Jun-17**	**Positive**	**MW118276**
UPE 069	*Rousettus aegyptiacus*	Population-level faecal	7-Jun-17	Negative	
UPE 070	*Rousettus aegyptiacus*	Population-level faecal	7-Jun-17	Negative	
**UPE 071**	***Rousettus aegyptiacus***	**Population-level faecal**	**7-Jun-17**	**Positive**	**MW118277**
UPE 072	*Rousettus aegyptiacus*	Population-level faecal	7-Jun-17	Negative	
UPE 073	*Rousettus aegyptiacus*	Population-level faecal	7-Jun-17	Negative	
**UPE 074**	***Rousettus aegyptiacus***	**Population-level faecal**	**7-Jun-17**	**Positive**	**MW118278**
UPE 075	*Rousettus aegyptiacus*	Population-level faecal	7-Jun-17	Negative	
UPE 076	*Rousettus aegyptiacus*	Population-level faecal	7-Jun-17	Negative	
UPE 077	*Rousettus aegyptiacus*	Population-level faecal	7-Jun-17	Negative	
UPE 102	*Rousettus aegyptiacus*	Population-level faecal	18-Jul-17	Negative	
UPE 104	*Rousettus aegyptiacus*	Population-level faecal	18-Jul-17	Negative	
UPE 105	*Rousettus aegyptiacus*	Population-level faecal	18-Jul-17	Negative	
UPE 106	*Rousettus aegyptiacus*	Population-level faecal	18-Jul-17	Negative	
UPE 107	*Rousettus aegyptiacus*	Population-level faecal	18-Jul-17	Negative	
UPE 108	*Rousettus aegyptiacus*	Population-level faecal	18-Jul-17	Negative	
**UPE 110**	***Rousettus aegyptiacus***	**Population-level faecal**	**18-Jul-17**	**Positive**	**MW118271**
UPE 129	*Rousettus aegyptiacus*	Population-level faecal	18-Jul-17	Negative	
UPE 130	*Rousettus aegyptiacus*	Population-level faecal	18-Jul-17	Negative	
UPE 134	*Rousettus aegyptiacus*	Population-level faecal	18-Jul-17	Negative	
UPE 135	*Rousettus aegyptiacus*	Population-level faecal	7-Aug-17	Negative	
UPE 136	*Rousettus aegyptiacus*	Population-level faecal	7-Aug-17	Negative	
UPE 137	*Rousettus aegyptiacus*	Population-level faecal	7-Aug-17	Negative	
UPE 138	*Rousettus aegyptiacus*	Population-level faecal	7-Aug-17	Negative	
UPE 139	*Rousettus aegyptiacus*	Population-level faecal	7-Aug-17	Negative	
UPE 140	*Rousettus aegyptiacus*	Population-level faecal	7-Aug-17	Negative	
UPE 141	*Rousettus aegyptiacus*	Population-level faecal	7-Aug-17	Negative	
UPE 142	*Rousettus aegyptiacus*	Population-level faecal	7-Aug-17	Negative	
UPE 143	*Rousettus aegyptiacus*	Population-level faecal	7-Aug-17	Negative	
UPE 144	*Rousettus aegyptiacus*	Population-level faecal	7-Aug-17	Negative	
UPE 145	*Rousettus aegyptiacus*	Population-level faecal	7-Aug-17	Negative	
UPE 146	*Rousettus aegyptiacus*	Population-level faecal	7-Aug-17	Negative	
UPE 147	*Rousettus aegyptiacus*	Population-level faecal	7-Aug-17	Negative	
UPE 148	*Rousettus aegyptiacus*	Population-level faecal	7-Aug-17	Negative	
UPE 149	*Rousettus aegyptiacus*	Population-level faecal	7-Aug-17	Negative	
UPE 150	*Rousettus aegyptiacus*	Population-level faecal	7-Aug-17	Negative	
**UPE 151**	***Rousettus aegyptiacus***	**Population-level faecal**	**7-Aug-17**	**Positive**	**MW118272**
UPE 152	*Rousettus aegyptiacus*	Population-level faecal	7-Aug-17	Negative	
UPE 153	*Rousettus aegyptiacus*	Population-level faecal	7-Aug-17	Negative	
**UPE 154**	***Rousettus aegyptiacus***	**Population-level faecal**	**7-Aug-17**	**Positive**	**MW118273**
**UPE 155**	***Rousettus aegyptiacus***	**Population-level faecal**	**7-Aug-17**	**Positive**	**MW118274**
UPE 156	*Rousettus aegyptiacus*	Population-level faecal	7-Aug-17	Negative	
UPE 157	*Rousettus aegyptiacus*	Population-level faecal	7-Aug-17	Negative	
UPE 158	*Rousettus aegyptiacus*	Population-level faecal	7-Aug-17	Negative	
UPE 159	*Rousettus aegyptiacus*	Population-level faecal	7-Aug-17	Negative	
UPE 160	*Rousettus aegyptiacus*	Population-level faecal	7-Aug-17	Negative	
UPE 206	*Rousettus aegyptiacus*	Population-level faecal	27-Sep-17	Negative	
**UPE 207**	***Rousettus aegyptiacus***	**Population-level faecal**	**27-Sep-17**	**Positive**	**OR365870**
UPE 208	*Rousettus aegyptiacus*	Population-level faecal	27-Sep-17	Negative	
UPE 209	*Rousettus aegyptiacus*	Population-level faecal	27-Sep-17	Negative	
**UPE 210**	***Rousettus aegyptiacus***	**Population-level faecal**	**27-Sep-17**	**Positive**	**OR365871**
UPE 211	*Rousettus aegyptiacus*	Population-level faecal	27-Sep-17	Negative	
**UPE 212**	***Rousettus aegyptiacus***	**Population-level faecal**	**27-Sep-17**	**Positive**	**OR365872**
UPE 213	*Rousettus aegyptiacus*	Population-level faecal	27-Sep-17	Negative	
**UPE 214**	***Rousettus aegyptiacus***	**Population-level faecal**	**27-Sep-17**	**Positive**	**OR365873**

Boldface: Population-level faecal samples which tested positive for rubulavirus RNA

Table S1 (*continued*): Egyptian rousette bat population-level faecal samples tested for rubulavirus RNA

Laboratory number	Source/Host	Sample type	Date collected	Result	Genbank Accession Number
UPE 215	*Rousettus aegyptiacus*	Population-level faecal	27-Sep-17	Negative	
UPE 216	*Rousettus aegyptiacus*	Population-level faecal	27-Sep-17	Negative	
UPE 217	*Rousettus aegyptiacus*	Population-level faecal	27-Sep-17	Negative	
UPE 218	*Rousettus aegyptiacus*	Population-level faecal	27-Sep-17	Negative	
UPE 219	*Rousettus aegyptiacus*	Population-level faecal	27-Sep-17	Negative	
UPE 221	*Rousettus aegyptiacus*	Population-level faecal	27-Sep-17	Negative	
UPE 222	*Rousettus aegyptiacus*	Population-level faecal	27-Sep-17	Negative	
UPE 223	*Rousettus aegyptiacus*	Population-level faecal	27-Sep-17	Negative	
**UPE 224**	***Rousettus aegyptiacus***	**Population-level faecal**	**27-Sep-17**	**Positive**	**OR365874**
UPE 225	*Rousettus aegyptiacus*	Population-level faecal	27-Sep-17	Negative	
UPE 226	*Rousettus aegyptiacus*	Population-level faecal	27-Sep-17	Negative	
UPE 227	*Rousettus aegyptiacus*	Population-level faecal	27-Sep-17	Negative	
UPE 228	*Rousettus aegyptiacus*	Population-level faecal	27-Sep-17	Negative	
**UPE 229**	***Rousettus aegyptiacus***	**Population-level faecal**	**27-Sep-17**	**Positive**	**OR365875**
UPE 230	*Rousettus aegyptiacus*	Population-level faecal	27-Sep-17	Negative	
UPE 231	*Rousettus aegyptiacus*	Population-level faecal	27-Sep-17	Negative	
UPE 232	*Rousettus aegyptiacus*	Population-level faecal	27-Sep-17	Negative	
UPE 233	*Rousettus aegyptiacus*	Population-level faecal	27-Sep-17	Negative	
UPE 234	*Rousettus aegyptiacus*	Population-level faecal	27-Sep-17	Negative	
UPE 235	*Rousettus aegyptiacus*	Population-level faecal	27-Sep-17	Negative	
**UPE 236**	***Rousettus aegyptiacus***	**Population-level faecal**	**27-Sep-17**	**Positive**	**OR365876**
UPE 237	*Rousettus aegyptiacus*	Population-level faecal	27-Sep-17	Negative	
UPE 238	*Rousettus aegyptiacus*	Population-level faecal	27-Sep-17	Negative	
UPE 239	*Rousettus aegyptiacus*	Population-level faecal	27-Sep-17	Negative	
UPE 240	*Rousettus aegyptiacus*	Population-level faecal	27-Sep-17	Negative	
UPE 241	*Rousettus aegyptiacus*	Population-level faecal	27-Sep-17	Negative	
UPE 242	*Rousettus aegyptiacus*	Population-level faecal	27-Sep-17	Negative	
**UPE 243**	***Rousettus aegyptiacus***	**Population-level faecal**	**27-Sep-17**	**Positive**	**OR365877**
UPE 244	*Rousettus aegyptiacus*	Population-level faecal	27-Sep-17	Negative	
UPE 245	*Rousettus aegyptiacus*	Population-level faecal	27-Sep-17	Negative	
UPE 246	*Rousettus aegyptiacus*	Population-level faecal	27-Sep-17	Negative	
UPE 274	*Rousettus aegyptiacus*	Population-level faecal	17-Oct-17	Negative	
UPE 275	*Rousettus aegyptiacus*	Population-level faecal	17-Oct-17	Negative	
UPE 276	*Rousettus aegyptiacus*	Population-level faecal	17-Oct-17	Negative	
UPE 277	*Rousettus aegyptiacus*	Population-level faecal	17-Oct-17	Negative	
UPE 278	*Rousettus aegyptiacus*	Population-level faecal	17-Oct-17	Negative	
UPE 279	*Rousettus aegyptiacus*	Population-level faecal	17-Oct-17	Negative	
UPE 280	*Rousettus aegyptiacus*	Population-level faecal	17-Oct-17	Negative	
UPE 281	*Rousettus aegyptiacus*	Population-level faecal	17-Oct-17	Negative	
UPE 282	*Rousettus aegyptiacus*	Population-level faecal	17-Oct-17	Negative	
UPE 283	*Rousettus aegyptiacus*	Population-level faecal	17-Oct-17	Negative	
UPE 284	*Rousettus aegyptiacus*	Population-level faecal	17-Oct-17	Negative	
UPE 285	*Rousettus aegyptiacus*	Population-level faecal	17-Oct-17	Negative	
UPE 286	*Rousettus aegyptiacus*	Population-level faecal	17-Oct-17	Negative	
UPE 287	*Rousettus aegyptiacus*	Population-level faecal	17-Oct-17	Negative	
UPE 288	*Rousettus aegyptiacus*	Population-level faecal	17-Oct-17	Negative	
UPE 289	*Rousettus aegyptiacus*	Population-level faecal	17-Oct-17	Negative	
UPE 290	*Rousettus aegyptiacus*	Population-level faecal	17-Oct-17	Negative	
UPE 291	*Rousettus aegyptiacus*	Population-level faecal	17-Oct-17	Negative	
UPE 292	*Rousettus aegyptiacus*	Population-level faecal	17-Oct-17	Negative	
UPE 293	*Rousettus aegyptiacus*	Population-level faecal	17-Oct-17	Negative	
**UPE 294**	***Rousettus aegyptiacus***	**Population-level faecal**	**17-Oct-17**	**Positive**	**OR365878**
UPE 295	*Rousettus aegyptiacus*	Population-level faecal	17-Oct-17	Negative	
UPE 296	*Rousettus aegyptiacus*	Population-level faecal	17-Oct-17	Negative	
UPE 297	*Rousettus aegyptiacus*	Population-level faecal	17-Oct-17	Negative	
UPE 298	*Rousettus aegyptiacus*	Population-level faecal	17-Oct-17	Negative	
UPE 299	*Rousettus aegyptiacus*	Population-level faecal	17-Oct-17	Negative	
UPE 300	*Rousettus aegyptiacus*	Population-level faecal	17-Oct-17	Negative	
UPE 301	*Rousettus aegyptiacus*	Population-level faecal	17-Oct-17	Negative	
UPE 302	*Rousettus aegyptiacus*	Population-level faecal	17-Oct-17	Negative	

Boldface: Population-level faecal samples which tested positive for rubulavirus RNA

Table S1 (*continued*): Egyptian rousette bat population-level faecal samples tested for rubulavirus RNA

Laboratory number	Source/Host	Sample type	Date collected	Result	Genbank Accession Number
UPE 303	*Rousettus aegyptiacus*	Population-level faecal	17-Oct-17	Negative	
UPE 304	*Rousettus aegyptiacus*	Population-level faecal	17-Oct-17	Negative	
UPE 305	*Rousettus aegyptiacus*	Population-level faecal	17-Oct-17	Negative	
UPE 306	*Rousettus aegyptiacus*	Population-level faecal	17-Oct-17	Negative	
UPE 307	*Rousettus aegyptiacus*	Population-level faecal	17-Oct-17	Negative	
UPE 308	*Rousettus aegyptiacus*	Population-level faecal	17-Oct-17	Negative	
UPE 309	*Rousettus aegyptiacus*	Population-level faecal	17-Oct-17	Negative	
**UPE 310**	***Rousettus aegyptiacus***	**Population-level faecal**	**17-Oct-17**	**Positive**	**OR365879**
UPE 311	*Rousettus aegyptiacus*	Population-level faecal	17-Oct-17	Negative	
UPE 368	*Rousettus aegyptiacus*	Population-level faecal	8-Nov-17	Negative	
UPE 369	*Rousettus aegyptiacus*	Population-level faecal	8-Nov-17	Negative	
UPE 370	*Rousettus aegyptiacus*	Population-level faecal	8-Nov-17	Negative	
UPE 371	*Rousettus aegyptiacus*	Population-level faecal	8-Nov-17	Negative	
UPE 372	*Rousettus aegyptiacus*	Population-level faecal	8-Nov-17	Negative	
UPE 373	*Rousettus aegyptiacus*	Population-level faecal	8-Nov-17	Negative	
UPE 375	*Rousettus aegyptiacus*	Population-level faecal	8-Nov-17	Negative	
UPE 376	*Rousettus aegyptiacus*	Population-level faecal	8-Nov-17	Negative	
UPE 377	*Rousettus aegyptiacus*	Population-level faecal	8-Nov-17	Negative	
UPE 378	*Rousettus aegyptiacus*	Population-level faecal	8-Nov-17	Negative	
UPE 379	*Rousettus aegyptiacus*	Population-level faecal	8-Nov-17	Negative	
UPE 380	*Rousettus aegyptiacus*	Population-level faecal	8-Nov-17	Negative	
UPE 381	*Rousettus aegyptiacus*	Population-level faecal	8-Nov-17	Negative	
UPE 382	*Rousettus aegyptiacus*	Population-level faecal	8-Nov-17	Negative	
UPE 383	*Rousettus aegyptiacus*	Population-level faecal	8-Nov-17	Negative	
UPE 384	*Rousettus aegyptiacus*	Population-level faecal	8-Nov-17	Negative	
**UPE 386**	***Rousettus aegyptiacus***	**Population-level faecal**	**8-Nov-17**	**Positive**	**OR365880**
UPE 387	*Rousettus aegyptiacus*	Population-level faecal	8-Nov-17	Negative	
UPE 388	*Rousettus aegyptiacus*	Population-level faecal	8-Nov-17	Negative	
UPE 389	*Rousettus aegyptiacus*	Population-level faecal	8-Nov-17	Negative	
UPE 390	*Rousettus aegyptiacus*	Population-level faecal	8-Nov-17	Negative	
UPE 391	*Rousettus aegyptiacus*	Population-level faecal	8-Nov-17	Negative	
UPE 392	*Rousettus aegyptiacus*	Population-level faecal	8-Nov-17	Negative	
UPE 393	*Rousettus aegyptiacus*	Population-level faecal	8-Nov-17	Negative	
UPE 394	*Rousettus aegyptiacus*	Population-level faecal	8-Nov-17	Negative	
UPE 395	*Rousettus aegyptiacus*	Population-level faecal	8-Nov-17	Negative	
UPE 396	*Rousettus aegyptiacus*	Population-level faecal	8-Nov-17	Negative	
UPE 397	*Rousettus aegyptiacus*	Population-level faecal	8-Nov-17	Negative	
UPE 398	*Rousettus aegyptiacus*	Population-level faecal	8-Nov-17	Negative	
UPE 399	*Rousettus aegyptiacus*	Population-level faecal	8-Nov-17	Negative	
UPE 400	*Rousettus aegyptiacus*	Population-level faecal	8-Nov-17	Negative	
UPE 402	*Rousettus aegyptiacus*	Population-level faecal	8-Nov-17	Negative	
UPE 403	*Rousettus aegyptiacus*	Population-level faecal	8-Nov-17	Negative	
UPE 404	*Rousettus aegyptiacus*	Population-level faecal	8-Nov-17	Negative	
UPE 405	*Rousettus aegyptiacus*	Population-level faecal	8-Nov-17	Negative	
UPE 427	*Rousettus aegyptiacus*	Population-level faecal	5-Dec-17	Negative	
UPE 428	*Rousettus aegyptiacus*	Population-level faecal	5-Dec-17	Negative	
UPE 429	*Rousettus aegyptiacus*	Population-level faecal	5-Dec-17	Negative	
UPE 430	*Rousettus aegyptiacus*	Population-level faecal	5-Dec-17	Negative	
UPE 431	*Rousettus aegyptiacus*	Population-level faecal	5-Dec-17	Negative	
UPE 432	*Rousettus aegyptiacus*	Population-level faecal	5-Dec-17	Negative	
UPE 433	*Rousettus aegyptiacus*	Population-level faecal	5-Dec-17	Negative	
UPE 434	*Rousettus aegyptiacus*	Population-level faecal	5-Dec-17	Negative	
UPE 435	*Rousettus aegyptiacus*	Population-level faecal	5-Dec-17	Negative	
UPE 436	*Rousettus aegyptiacus*	Population-level faecal	5-Dec-17	Negative	
UPE 437	*Rousettus aegyptiacus*	Population-level faecal	5-Dec-17	Negative	
UPE 438	*Rousettus aegyptiacus*	Population-level faecal	5-Dec-17	Negative	
UPE 439	*Rousettus aegyptiacus*	Population-level faecal	5-Dec-17	Negative	
UPE 440	*Rousettus aegyptiacus*	Population-level faecal	5-Dec-17	Negative	
UPE 441	*Rousettus aegyptiacus*	Population-level faecal	5-Dec-17	Negative	
UPE 442	*Rousettus aegyptiacus*	Population-level faecal	5-Dec-17	Negative	
UPE 443	*Rousettus aegyptiacus*	Population-level faecal	5-Dec-17	Negative	

Boldface: Population-level faecal samples which tested positive for rubulavirus RNA

Table S1 (*continued*): Egyptian rousette bat population-level faecal samples tested for rubulavirus RNA

Laboratory number	Source/Host	Sample type	Date collected	Result	Genbank Accession Number
UPE 444	*Rousettus aegyptiacus*	Population-level faecal	5-Dec-17	Negative	
UPE 445	*Rousettus aegyptiacus*	Population-level faecal	5-Dec-17	Negative	
UPE 446	*Rousettus aegyptiacus*	Population-level faecal	5-Dec-17	Negative	
UPE 447	*Rousettus aegyptiacus*	Population-level faecal	5-Dec-17	Negative	
UPE 448	*Rousettus aegyptiacus*	Population-level faecal	5-Dec-17	Negative	
UPE 449	*Rousettus aegyptiacus*	Population-level faecal	5-Dec-17	Negative	
UPE 451	*Rousettus aegyptiacus*	Population-level faecal	5-Dec-17	Negative	
UPE 452	*Rousettus aegyptiacus*	Population-level faecal	5-Dec-17	Negative	
UPE 453	*Rousettus aegyptiacus*	Population-level faecal	5-Dec-17	Negative	
UPE 454	*Rousettus aegyptiacus*	Population-level faecal	5-Dec-17	Negative	
UPE 455	*Rousettus aegyptiacus*	Population-level faecal	5-Dec-17	Negative	
UPE 456	*Rousettus aegyptiacus*	Population-level faecal	5-Dec-17	Negative	
UPE 457	*Rousettus aegyptiacus*	Population-level faecal	5-Dec-17	Negative	
UPE 490	*Rousettus aegyptiacus*	Population-level faecal	5-Dec-17	Negative	
UPE 491	*Rousettus aegyptiacus*	Population-level faecal	5-Dec-17	Negative	
UPE 492	*Rousettus aegyptiacus*	Population-level faecal	5-Dec-17	Negative	
UPE 493	*Rousettus aegyptiacus*	Population-level faecal	5-Dec-17	Negative	
UPE 494	*Rousettus aegyptiacus*	Population-level faecal	5-Dec-17	Negative	
UPE 495	*Rousettus aegyptiacus*	Population-level faecal	5-Dec-17	Negative	
UPE 496	*Rousettus aegyptiacus*	Population-level faecal	5-Dec-17	Negative	
UPE 497	*Rousettus aegyptiacus*	Population-level faecal	5-Dec-17	Negative	
UPE 498	*Rousettus aegyptiacus*	Population-level faecal	5-Dec-17	Negative	
UPE 538	*Rousettus aegyptiacus*	Population-level faecal	24-Jan-18	Negative	
UPE 539	*Rousettus aegyptiacus*	Population-level faecal	24-Jan-18	Negative	
UPE 540	*Rousettus aegyptiacus*	Population-level faecal	24-Jan-18	Negative	
**UPE 541**	***Rousettus aegyptiacus***	**Population-level faecal**	**24-Jan-18**	**Positive**	**OR365881**
UPE 542	*Rousettus aegyptiacus*	Population-level faecal	24-Jan-18	Negative	
UPE 543	*Rousettus aegyptiacus*	Population-level faecal	24-Jan-18	Negative	
UPE 544	*Rousettus aegyptiacus*	Population-level faecal	24-Jan-18	Negative	
UPE 545	*Rousettus aegyptiacus*	Population-level faecal	24-Jan-18	Negative	
UPE 546	*Rousettus aegyptiacus*	Population-level faecal	24-Jan-18	Negative	
UPE 547	*Rousettus aegyptiacus*	Population-level faecal	24-Jan-18	Negative	
UPE 548	*Rousettus aegyptiacus*	Population-level faecal	24-Jan-18	Negative	
UPE 549	*Rousettus aegyptiacus*	Population-level faecal	24-Jan-18	Negative	
UPE 550	*Rousettus aegyptiacus*	Population-level faecal	24-Jan-18	Negative	
UPE 551	*Rousettus aegyptiacus*	Population-level faecal	24-Jan-18	Negative	
UPE 552	*Rousettus aegyptiacus*	Population-level faecal	24-Jan-18	Negative	
UPE 553	*Rousettus aegyptiacus*	Population-level faecal	24-Jan-18	Negative	
UPE 554	*Rousettus aegyptiacus*	Population-level faecal	24-Jan-18	Negative	
UPE 555	*Rousettus aegyptiacus*	Population-level faecal	24-Jan-18	Negative	
UPE 556	*Rousettus aegyptiacus*	Population-level faecal	24-Jan-18	Negative	
UPE 557	*Rousettus aegyptiacus*	Population-level faecal	24-Jan-18	Negative	
UPE 558	*Rousettus aegyptiacus*	Population-level faecal	24-Jan-18	Negative	
UPE 559	*Rousettus aegyptiacus*	Population-level faecal	24-Jan-18	Negative	
UPE 560	*Rousettus aegyptiacus*	Population-level faecal	24-Jan-18	Negative	
UPE 561	*Rousettus aegyptiacus*	Population-level faecal	24-Jan-18	Negative	
UPE 562	*Rousettus aegyptiacus*	Population-level faecal	24-Jan-18	Negative	
**UPE 563**	***Rousettus aegyptiacus***	**Population-level faecal**	**24-Jan-18**	**Positive**	**OR365882**
UPE 564	*Rousettus aegyptiacus*	Population-level faecal	24-Jan-18	Negative	
UPE 565	*Rousettus aegyptiacus*	Population-level faecal	24-Jan-18	Negative	
UPE 566	*Rousettus aegyptiacus*	Population-level faecal	24-Jan-18	Negative	
UPE 567	*Rousettus aegyptiacus*	Population-level faecal	24-Jan-18	Negative	
UPE 568	*Rousettus aegyptiacus*	Population-level faecal	24-Jan-18	Negative	
UPE 569	*Rousettus aegyptiacus*	Population-level faecal	24-Jan-18	Negative	
UPE 570	*Rousettus aegyptiacus*	Population-level faecal	24-Jan-18	Negative	
UPE 571	*Rousettus aegyptiacus*	Population-level faecal	24-Jan-18	Negative	
UPE 572	*Rousettus aegyptiacus*	Population-level faecal	24-Jan-18	Negative	
UPE 573	*Rousettus aegyptiacus*	Population-level faecal	24-Jan-18	Negative	
UPE 632	*Rousettus aegyptiacus*	Population-level faecal	7-Feb-18	Negative	
UPE 633	*Rousettus aegyptiacus*	Population-level faecal	7-Feb-18	Negative	
UPE 634	*Rousettus aegyptiacus*	Population-level faecal	7-Feb-18	Negative	
UPE 635	*Rousettus aegyptiacus*	Population-level faecal	7-Feb-18	Negative	
UPE 636	*Rousettus aegyptiacus*	Population-level faecal	7-Feb-18	Negative	

Boldface: Population-level faecal samples which tested positive for rubulavirus RNA

Table S1 (*continued*)**:** Egyptian rousette bat population-level faecal samples tested for rubulavirus RNA

Laboratory number	Source/Host	Sample type	Date collected	Result	Genbank Accession Number
UPE 637	*Rousettus aegyptiacus*	Population-level faecal	7-Feb-18	Negative	
UPE 638	*Rousettus aegyptiacus*	Population-level faecal	7-Feb-18	Negative	
UPE 639	*Rousettus aegyptiacus*	Population-level faecal	7-Feb-18	Negative	
UPE 640	*Rousettus aegyptiacus*	Population-level faecal	7-Feb-18	Negative	
UPE 641	*Rousettus aegyptiacus*	Population-level faecal	7-Feb-18	Negative	
UPE 642	*Rousettus aegyptiacus*	Population-level faecal	7-Feb-18	Negative	
UPE 643	*Rousettus aegyptiacus*	Population-level faecal	7-Feb-18	Negative	
UPE 644	*Rousettus aegyptiacus*	Population-level faecal	7-Feb-18	Negative	
UPE 645	*Rousettus aegyptiacus*	Population-level faecal	7-Feb-18	Negative	
UPE 646	*Rousettus aegyptiacus*	Population-level faecal	7-Feb-18	Negative	
UPE 647	*Rousettus aegyptiacus*	Population-level faecal	7-Feb-18	Negative	
UPE 648	*Rousettus aegyptiacus*	Population-level faecal	7-Feb-18	Negative	
UPE 649	*Rousettus aegyptiacus*	Population-level faecal	7-Feb-18	Negative	
UPE 650	*Rousettus aegyptiacus*	Population-level faecal	7-Feb-18	Negative	
UPE 651	*Rousettus aegyptiacus*	Population-level faecal	7-Feb-18	Negative	
UPE 652	*Rousettus aegyptiacus*	Population-level faecal	7-Feb-18	Negative	
UPE 653	*Rousettus aegyptiacus*	Population-level faecal	7-Feb-18	Negative	
UPE 654	*Rousettus aegyptiacus*	Population-level faecal	7-Feb-18	Negative	
UPE 655	*Rousettus aegyptiacus*	Population-level faecal	7-Feb-18	Negative	
UPE 656	*Rousettus aegyptiacus*	Population-level faecal	7-Feb-18	Negative	
UPE 657	*Rousettus aegyptiacus*	Population-level faecal	7-Feb-18	Negative	
UPE 658	*Rousettus aegyptiacus*	Population-level faecal	7-Feb-18	Negative	
UPE 659	*Rousettus aegyptiacus*	Population-level faecal	7-Feb-18	Negative	
UPE 660	*Rousettus aegyptiacus*	Population-level faecal	7-Feb-18	Negative	
UPE 661	*Rousettus aegyptiacus*	Population-level faecal	7-Feb-18	Negative	
UPE 662	*Rousettus aegyptiacus*	Population-level faecal	7-Feb-18	Negative	
UPE 663	*Rousettus aegyptiacus*	Population-level faecal	7-Feb-18	Negative	
UPE 664	*Rousettus aegyptiacus*	Population-level faecal	7-Feb-18	Negative	
UPE 665	*Rousettus aegyptiacus*	Population-level faecal	7-Feb-18	Negative	
UPE 666	*Rousettus aegyptiacus*	Population-level faecal	7-Feb-18	Negative	
UPE 667	*Rousettus aegyptiacus*	Population-level faecal	7-Feb-18	Negative	
UPE 668	*Rousettus aegyptiacus*	Population-level faecal	7-Feb-18	Negative	
UPE 692	*Rousettus aegyptiacus*	Population-level faecal	6-Mar-18	Negative	
UPE 693	*Rousettus aegyptiacus*	Population-level faecal	6-Mar-18	Negative	
UPE 694	*Rousettus aegyptiacus*	Population-level faecal	6-Mar-18	Negative	
UPE 695	*Rousettus aegyptiacus*	Population-level faecal	6-Mar-18	Negative	
**UPE 696**	***Rousettus aegyptiacus***	**Population-level faecal**	**6-Mar-18**	**Positive**	**OR365883**
**UPE 697**	***Rousettus aegyptiacus***	**Population-level faecal**	**6-Mar-18**	**Positive**	**OR365884**
**UPE 698**	***Rousettus aegyptiacus***	**Population-level faecal**	**6-Mar-18**	**Positive**	**OR365885**
UPE 699	*Rousettus aegyptiacus*	Population-level faecal	6-Mar-18	Negative	
UPE 700	*Rousettus aegyptiacus*	Population-level faecal	6-Mar-18	Negative	
UPE 701	*Rousettus aegyptiacus*	Population-level faecal	6-Mar-18	Negative	
UPE 702	*Rousettus aegyptiacus*	Population-level faecal	6-Mar-18	Negative	
UPE 703	*Rousettus aegyptiacus*	Population-level faecal	6-Mar-18	Negative	
UPE 704	*Rousettus aegyptiacus*	Population-level faecal	6-Mar-18	Negative	
UPE 705	*Rousettus aegyptiacus*	Population-level faecal	6-Mar-18	Negative	
UPE 706	*Rousettus aegyptiacus*	Population-level faecal	6-Mar-18	Negative	
UPE 707	*Rousettus aegyptiacus*	Population-level faecal	6-Mar-18	Negative	
UPE 708	*Rousettus aegyptiacus*	Population-level faecal	6-Mar-18	Negative	
UPE 709	*Rousettus aegyptiacus*	Population-level faecal	6-Mar-18	Negative	
UPE 710	*Rousettus aegyptiacus*	Population-level faecal	6-Mar-18	Negative	
UPE 711	*Rousettus aegyptiacus*	Population-level faecal	6-Mar-18	Negative	
UPE 712	*Rousettus aegyptiacus*	Population-level faecal	6-Mar-18	Negative	
UPE 713	*Rousettus aegyptiacus*	Population-level faecal	6-Mar-18	Negative	
UPE 714	*Rousettus aegyptiacus*	Population-level faecal	6-Mar-18	Negative	
UPE 715	*Rousettus aegyptiacus*	Population-level faecal	6-Mar-18	Negative	
UPE 716	*Rousettus aegyptiacus*	Population-level faecal	6-Mar-18	Negative	
UPE 717	*Rousettus aegyptiacus*	Population-level faecal	6-Mar-18	Negative	
UPE 718	*Rousettus aegyptiacus*	Population-level faecal	6-Mar-18	Negative	
UPE 719	*Rousettus aegyptiacus*	Population-level faecal	6-Mar-18	Negative	
UPE 720	*Rousettus aegyptiacus*	Population-level faecal	6-Mar-18	Negative	
UPE 721	*Rousettus aegyptiacus*	Population-level faecal	6-Mar-18	Negative	

Boldface: Population-level faecal samples which tested positive for rubulavirus RNA

Table S1 (*continued*): Egyptian rousette bat population-level faecal samples tested for rubulavirus RNA

Laboratory number	Source/Host	Sample type	Date collected	Result	Genbank Accession Number
UPE 734	*Rousettus aegyptiacus*	Population-level faecal	11-Apr-18	Negative	
UPE 735	*Rousettus aegyptiacus*	Population-level faecal	11-Apr-18	Negative	
UPE 736	*Rousettus aegyptiacus*	Population-level faecal	11-Apr-18	Negative	
UPE 739	*Rousettus aegyptiacus*	Population-level faecal	11-Apr-18	Negative	
UPE 740	*Rousettus aegyptiacus*	Population-level faecal	11-Apr-18	Negative	
UPE 741	*Rousettus aegyptiacus*	Population-level faecal	11-Apr-18	Negative	
UPE 742	*Rousettus aegyptiacus*	Population-level faecal	11-Apr-18	Negative	
UPE 743	*Rousettus aegyptiacus*	Population-level faecal	11-Apr-18	Negative	
UPE 744	*Rousettus aegyptiacus*	Population-level faecal	11-Apr-18	Negative	
UPE 746	*Rousettus aegyptiacus*	Population-level faecal	11-Apr-18	Negative	
UPE 747	*Rousettus aegyptiacus*	Population-level faecal	11-Apr-18	Negative	
UPE 748	*Rousettus aegyptiacus*	Population-level faecal	11-Apr-18	Negative	
UPE 749	*Rousettus aegyptiacus*	Population-level faecal	11-Apr-18	Negative	
**UPE 750**	***Rousettus aegyptiacus***	**Population-level faecal**	**11-Apr-18**	**Positive**	**OR365886**
**UPE 751**	***Rousettus aegyptiacus***	**Population-level faecal**	**11-Apr-18**	**Positive**	**OR365887**
UPE 752	*Rousettus aegyptiacus*	Population-level faecal	11-Apr-18	Negative	
UPE 753	*Rousettus aegyptiacus*	Population-level faecal	11-Apr-18	Negative	
UPE 754	*Rousettus aegyptiacus*	Population-level faecal	11-Apr-18	Negative	
UPE 756	*Rousettus aegyptiacus*	Population-level faecal	11-Apr-18	Negative	
UPE 757	*Rousettus aegyptiacus*	Population-level faecal	11-Apr-18	Negative	
UPE 758	*Rousettus aegyptiacus*	Population-level faecal	11-Apr-18	Negative	
UPE 770	*Rousettus aegyptiacus*	Population-level faecal	9-May-18	Negative	
UPE 771	*Rousettus aegyptiacus*	Population-level faecal	9-May-18	Negative	
UPE 772	*Rousettus aegyptiacus*	Population-level faecal	9-May-18	Negative	
UPE 773	*Rousettus aegyptiacus*	Population-level faecal	9-May-18	Negative	
UPE 774	*Rousettus aegyptiacus*	Population-level faecal	9-May-18	Negative	
UPE 775	*Rousettus aegyptiacus*	Population-level faecal	9-May-18	Negative	
**UPE 776**	***Rousettus aegyptiacus***	**Population-level faecal**	**9-May-18**	**Positive**	**OR365888**
UPE 777	*Rousettus aegyptiacus*	Population-level faecal	9-May-18	Negative	
UPE 778	*Rousettus aegyptiacus*	Population-level faecal	9-May-18	Negative	
UPE 779	*Rousettus aegyptiacus*	Population-level faecal	9-May-18	Negative	
**UPE 780**	***Rousettus aegyptiacus***	**Population-level faecal**	**9-May-18**	**Positive**	**OR365889**
**UPE 781**	***Rousettus aegyptiacus***	**Population-level faecal**	**9-May-18**	**Positive**	**OR365890**
UPE 782	*Rousettus aegyptiacus*	Population-level faecal	9-May-18	Negative	
UPE 783	*Rousettus aegyptiacus*	Population-level faecal	9-May-18	Negative	
UPE 784	*Rousettus aegyptiacus*	Population-level faecal	9-May-18	Negative	
UPE 791	*Rousettus aegyptiacus*	Population-level faecal	6-Jun-18	Negative	
UPE 793	*Rousettus aegyptiacus*	Population-level faecal	6-Jun-18	Negative	
UPE 794	*Rousettus aegyptiacus*	Population-level faecal	6-Jun-18	Negative	
UPE 795	*Rousettus aegyptiacus*	Population-level faecal	6-Jun-18	Negative	
UPE 796	*Rousettus aegyptiacus*	Population-level faecal	6-Jun-18	Negative	
UPE 797	*Rousettus aegyptiacus*	Population-level faecal	6-Jun-18	Negative	
UPE 819	*Rousettus aegyptiacus*	Population-level faecal	3-Jul-18	Negative	
UPE 820	*Rousettus aegyptiacus*	Population-level faecal	3-Jul-18	Negative	
UPE 821	*Rousettus aegyptiacus*	Population-level faecal	3-Jul-18	Negative	
UPE 822	*Rousettus aegyptiacus*	Population-level faecal	3-Jul-18	Negative	
UPE 823	*Rousettus aegyptiacus*	Population-level faecal	3-Jul-18	Negative	
UPE 824	*Rousettus aegyptiacus*	Population-level faecal	3-Jul-18	Negative	
UPE 825	*Rousettus aegyptiacus*	Population-level faecal	3-Jul-18	Negative	
UPE 830	*Rousettus aegyptiacus*	Population-level faecal	3-Jul-18	Negative	
UPE 832	*Rousettus aegyptiacus*	Population-level faecal	1-Aug-18	Negative	
UPE 833	*Rousettus aegyptiacus*	Population-level faecal	1-Aug-18	Negative	
UPE 834	*Rousettus aegyptiacus*	Population-level faecal	1-Aug-18	Negative	
UPE 835	*Rousettus aegyptiacus*	Population-level faecal	1-Aug-18	Negative	
UPE 836	*Rousettus aegyptiacus*	Population-level faecal	1-Aug-18	Negative	
UPE 837	*Rousettus aegyptiacus*	Population-level faecal	1-Aug-18	Negative	
UPE 838	*Rousettus aegyptiacus*	Population-level faecal	1-Aug-18	Negative	
UPE 904	*Rousettus aegyptiacus*	Population-level faecal	5-Sep-18	Negative	
UPE 905	*Rousettus aegyptiacus*	Population-level faecal	5-Sep-18	Negative	
UPE 906	*Rousettus aegyptiacus*	Population-level faecal	5-Sep-18	Negative	
UPE 907	*Rousettus aegyptiacus*	Population-level faecal	5-Sep-18	Negative	
UPE 908	*Rousettus aegyptiacus*	Population-level faecal	5-Sep-18	Negative	

Boldface: Population-level faecal samples which tested positive for rubulavirus RNA

Table S1 (*continued*): Egyptian rousette bat population-level faecal samples tested for rubulavirus RNA

Laboratory number	Source/Host	Sample type	Date collected	Result	Genbank Accession Number
UPE 909	*Rousettus aegyptiacus*	Population-level faecal	5-Sep-18	Negative	
UPE 910	*Rousettus aegyptiacus*	Population-level faecal	5-Sep-18	Negative	
UPE 911	*Rousettus aegyptiacus*	Population-level faecal	5-Sep-18	Negative	
UPE 912	*Rousettus aegyptiacus*	Population-level faecal	5-Sep-18	Negative	
UPE 913	*Rousettus aegyptiacus*	Population-level faecal	5-Sep-18	Negative	
UPE 914	*Rousettus aegyptiacus*	Population-level faecal	5-Sep-18	Negative	
UPE 915	*Rousettus aegyptiacus*	Population-level faecal	5-Sep-18	Negative	
UPE 916	*Rousettus aegyptiacus*	Population-level faecal	5-Sep-18	Negative	
UPE 917	*Rousettus aegyptiacus*	Population-level faecal	5-Sep-18	Negative	
UPE 918	*Rousettus aegyptiacus*	Population-level faecal	5-Sep-18	Negative	
UPE 919	*Rousettus aegyptiacus*	Population-level faecal	5-Sep-18	Negative	
UPE 920	*Rousettus aegyptiacus*	Population-level faecal	5-Sep-18	Negative	
UPE 921	*Rousettus aegyptiacus*	Population-level faecal	5-Sep-18	Negative	
UPE 922	*Rousettus aegyptiacus*	Population-level faecal	5-Sep-18	Negative	
UPE 923	*Rousettus aegyptiacus*	Population-level faecal	5-Sep-18	Negative	
UPE 924	*Rousettus aegyptiacus*	Population-level faecal	5-Sep-18	Negative	
UPE 925	*Rousettus aegyptiacus*	Population-level faecal	5-Sep-18	Negative	
UPE 926	*Rousettus aegyptiacus*	Population-level faecal	5-Sep-18	Negative	
UPE 927	*Rousettus aegyptiacus*	Population-level faecal	5-Sep-18	Negative	
UPE 928	*Rousettus aegyptiacus*	Population-level faecal	5-Sep-18	Negative	
UPE 929	*Rousettus aegyptiacus*	Population-level faecal	5-Sep-18	Negative	
UPE 930	*Rousettus aegyptiacus*	Population-level faecal	5-Sep-18	Negative	
UPE 931	*Rousettus aegyptiacus*	Population-level faecal	5-Sep-18	Negative	
UPE 932	*Rousettus aegyptiacus*	Population-level faecal	5-Sep-18	Negative	
UPE 933	*Rousettus aegyptiacus*	Population-level faecal	5-Sep-18	Negative	
UPE 995	*Rousettus aegyptiacus*	Population-level faecal	2-Oct-18	Negative	
UPE 996	*Rousettus aegyptiacus*	Population-level faecal	2-Oct-18	Negative	
UPE 997	*Rousettus aegyptiacus*	Population-level faecal	2-Oct-18	Negative	
UPE 998	*Rousettus aegyptiacus*	Population-level faecal	2-Oct-18	Negative	
UPE 999	*Rousettus aegyptiacus*	Population-level faecal	2-Oct-18	Negative	
UPE 1000	*Rousettus aegyptiacus*	Population-level faecal	2-Oct-18	Negative	
**UPE 1001**	***Rousettus aegyptiacus***	**Population-level faecal**	**2-Oct-18**	**Positive**	**OR365891**
UPE 1002	*Rousettus aegyptiacus*	Population-level faecal	2-Oct-18	Negative	
UPE 1003	*Rousettus aegyptiacus*	Population-level faecal	2-Oct-18	Negative	
UPE 1004	*Rousettus aegyptiacus*	Population-level faecal	2-Oct-18	Negative	
UPE 1005	*Rousettus aegyptiacus*	Population-level faecal	2-Oct-18	Negative	
UPE 1006	*Rousettus aegyptiacus*	Population-level faecal	2-Oct-18	Negative	
**UPE 1007**	***Rousettus aegyptiacus***	**Population-level faecal**	**2-Oct-18**	**Positive**	**OR365892**
UPE 1008	*Rousettus aegyptiacus*	Population-level faecal	2-Oct-18	Negative	
UPE 1009	*Rousettus aegyptiacus*	Population-level faecal	2-Oct-18	Negative	
UPE 1010	*Rousettus aegyptiacus*	Population-level faecal	2-Oct-18	Negative	
UPE 1011	*Rousettus aegyptiacus*	Population-level faecal	2-Oct-18	Negative	
UPE 1012	*Rousettus aegyptiacus*	Population-level faecal	2-Oct-18	Negative	
UPE 1013	*Rousettus aegyptiacus*	Population-level faecal	2-Oct-18	Negative	
UPE 1014	*Rousettus aegyptiacus*	Population-level faecal	2-Oct-18	Negative	
UPE 1015	*Rousettus aegyptiacus*	Population-level faecal	2-Oct-18	Negative	
**UPE 1016**	***Rousettus aegyptiacus***	**Population-level faecal**	**2-Oct-18**	**Positive**	**OR365893**
UPE 1017	*Rousettus aegyptiacus*	Population-level faecal	2-Oct-18	Negative	
UPE 1018	*Rousettus aegyptiacus*	Population-level faecal	2-Oct-18	Negative	
UPE 1019	*Rousettus aegyptiacus*	Population-level faecal	2-Oct-18	Negative	
UPE 1020	*Rousettus aegyptiacus*	Population-level faecal	2-Oct-18	Negative	
UPE 1021	*Rousettus aegyptiacus*	Population-level faecal	2-Oct-18	Negative	
UPE 1022	*Rousettus aegyptiacus*	Population-level faecal	2-Oct-18	Negative	
UPE 1023	*Rousettus aegyptiacus*	Population-level faecal	2-Oct-18	Negative	
**UPE 1024**	***Rousettus aegyptiacus***	**Population-level faecal**	**2-Oct-18**	**Positive**	**OR365894**
UPE 1025	*Rousettus aegyptiacus*	Population-level faecal	2-Oct-18	Negative	
UPE 1026	*Rousettus aegyptiacus*	Population-level faecal	2-Oct-18	Negative	
UPE 1027	*Rousettus aegyptiacus*	Population-level faecal	2-Oct-18	Negative	
UPE 1028	*Rousettus aegyptiacus*	Population-level faecal	2-Oct-18	Negative	
UPE 1029	*Rousettus aegyptiacus*	Population-level faecal	2-Oct-18	Negative	
UPE 1095	*Rousettus aegyptiacus*	Population-level faecal	7-Nov-18	Negative	

Boldface: Population-level faecal samples which tested positive for rubulavirus RNA

Table S1 (*continued*): Egyptian rousette bat population-level faecal samples tested for rubulavirus RNA

Laboratory number	Source/Host	Sample type	Date collected	Result	Genbank Accession Number
UPE 1096	*Rousettus aegyptiacus*	Population-level faecal	7-Nov-18	Negative	
UPE 1097	*Rousettus aegyptiacus*	Population-level faecal	7-Nov-18	Negative	
UPE 1098	*Rousettus aegyptiacus*	Population-level faecal	7-Nov-18	Negative	
UPE 1099	*Rousettus aegyptiacus*	Population-level faecal	7-Nov-18	Negative	
UPE 1100	*Rousettus aegyptiacus*	Population-level faecal	7-Nov-18	Negative	
UPE 1101	*Rousettus aegyptiacus*	Population-level faecal	7-Nov-18	Negative	
UPE 1102	*Rousettus aegyptiacus*	Population-level faecal	7-Nov-18	Negative	
UPE 1103	*Rousettus aegyptiacus*	Population-level faecal	7-Nov-18	Negative	
UPE 1104	*Rousettus aegyptiacus*	Population-level faecal	7-Nov-18	Negative	
UPE 1105	*Rousettus aegyptiacus*	Population-level faecal	7-Nov-18	Negative	
UPE 1106	*Rousettus aegyptiacus*	Population-level faecal	7-Nov-18	Negative	
UPE 1107	*Rousettus aegyptiacus*	Population-level faecal	7-Nov-18	Negative	
UPE 1108	*Rousettus aegyptiacus*	Population-level faecal	7-Nov-18	Negative	
UPE 1109	*Rousettus aegyptiacus*	Population-level faecal	7-Nov-18	Negative	
UPE 1110	*Rousettus aegyptiacus*	Population-level faecal	7-Nov-18	Negative	
UPE 1111	*Rousettus aegyptiacus*	Population-level faecal	7-Nov-18	Negative	
UPE 1112	*Rousettus aegyptiacus*	Population-level faecal	7-Nov-18	Negative	
UPE 1113	*Rousettus aegyptiacus*	Population-level faecal	7-Nov-18	Negative	
UPE 1114	*Rousettus aegyptiacus*	Population-level faecal	7-Nov-18	Negative	
UPE 1115	*Rousettus aegyptiacus*	Population-level faecal	7-Nov-18	Negative	
UPE 1116	*Rousettus aegyptiacus*	Population-level faecal	7-Nov-18	Negative	
UPE 1117	*Rousettus aegyptiacus*	Population-level faecal	7-Nov-18	Negative	
UPE 1118	*Rousettus aegyptiacus*	Population-level faecal	7-Nov-18	Negative	
UPE 1119	*Rousettus aegyptiacus*	Population-level faecal	7-Nov-18	Negative	
UPE 1120	*Rousettus aegyptiacus*	Population-level faecal	7-Nov-18	Negative	
UPE 1121	*Rousettus aegyptiacus*	Population-level faecal	7-Nov-18	Negative	
UPE 1122	*Rousettus aegyptiacus*	Population-level faecal	7-Nov-18	Negative	
UPE 1123	*Rousettus aegyptiacus*	Population-level faecal	7-Nov-18	Negative	
UPE 1124	*Rousettus aegyptiacus*	Population-level faecal	7-Nov-18	Negative	
UPE 1125	*Rousettus aegyptiacus*	Population-level faecal	7-Nov-18	Negative	
UPE 1126	*Rousettus aegyptiacus*	Population-level faecal	7-Nov-18	Negative	
UPE 1127	*Rousettus aegyptiacus*	Population-level faecal	7-Nov-18	Negative	
UPE 1128	*Rousettus aegyptiacus*	Population-level faecal	7-Nov-18	Negative	
UPE 1129	*Rousettus aegyptiacus*	Population-level faecal	7-Nov-18	Negative	
UPE 1130	*Rousettus aegyptiacus*	Population-level faecal	7-Nov-18	Negative	
UPE 1131	*Rousettus aegyptiacus*	Population-level faecal	7-Nov-18	Negative	
UPE 1132	*Rousettus aegyptiacus*	Population-level faecal	7-Nov-18	Negative	
UPE 1133	*Rousettus aegyptiacus*	Population-level faecal	7-Nov-18	Negative	
UPE 1134	*Rousettus aegyptiacus*	Population-level faecal	7-Nov-18	Negative	
UPE 1135	*Rousettus aegyptiacus*	Population-level faecal	7-Nov-18	Negative	
UPE 1287	*Rousettus aegyptiacus*	Population-level faecal	4-Dec-18	Negative	
UPE 1288	*Rousettus aegyptiacus*	Population-level faecal	4-Dec-18	Negative	
UPE 1289	*Rousettus aegyptiacus*	Population-level faecal	4-Dec-18	Negative	
UPE 1290	*Rousettus aegyptiacus*	Population-level faecal	4-Dec-18	Negative	
UPE 1291	*Rousettus aegyptiacus*	Population-level faecal	4-Dec-18	Negative	
UPE 1292	*Rousettus aegyptiacus*	Population-level faecal	4-Dec-18	Negative	
UPE 1293	*Rousettus aegyptiacus*	Population-level faecal	4-Dec-18	Negative	
UPE 1294	*Rousettus aegyptiacus*	Population-level faecal	4-Dec-18	Negative	
UPE 1295	*Rousettus aegyptiacus*	Population-level faecal	4-Dec-18	Negative	
UPE 1296	*Rousettus aegyptiacus*	Population-level faecal	4-Dec-18	Negative	
UPE 1297	*Rousettus aegyptiacus*	Population-level faecal	4-Dec-18	Negative	
UPE 1298	*Rousettus aegyptiacus*	Population-level faecal	4-Dec-18	Negative	
UPE 1299	*Rousettus aegyptiacus*	Population-level faecal	4-Dec-18	Negative	
UPE 1300	*Rousettus aegyptiacus*	Population-level faecal	4-Dec-18	Negative	
UPE 1301	*Rousettus aegyptiacus*	Population-level faecal	4-Dec-18	Negative	
UPE 1302	*Rousettus aegyptiacus*	Population-level faecal	4-Dec-18	Negative	
UPE 1303	*Rousettus aegyptiacus*	Population-level faecal	4-Dec-18	Negative	
UPE 1304	*Rousettus aegyptiacus*	Population-level faecal	4-Dec-18	Negative	
UPE 1305	*Rousettus aegyptiacus*	Population-level faecal	4-Dec-18	Negative	
UPE 1306	*Rousettus aegyptiacus*	Population-level faecal	4-Dec-18	Negative	
UPE 1307	*Rousettus aegyptiacus*	Population-level faecal	4-Dec-18	Negative	

UPE 1308	*Rousettus aegyptiacus*	Population-level faecal	4-Dec-18	Negative	

Boldface: Population-level faecal samples which tested positive for rubulavirus RNA

Table S1 (*continued*): Egyptian rousette bat population-level faecal samples tested for rubulavirus RNA

**Laboratory number**	**Source/Host**	**Sample type**	**Date collected**	**Result**	**Genbank Accession Number**
UPE 1309	*Rousettus aegyptiacus*	Population-level faecal	4-Dec-18	Negative	
UPE 1310	*Rousettus aegyptiacus*	Population-level faecal	4-Dec-18	Negative	
UPE 1311	*Rousettus aegyptiacus*	Population-level faecal	4-Dec-18	Negative	
UPE 1314	*Rousettus aegyptiacus*	Population-level faecal	4-Dec-18	Negative	
UPE 1315	*Rousettus aegyptiacus*	Population-level faecal	4-Dec-18	Negative	
UPE 1316	*Rousettus aegyptiacus*	Population-level faecal	4-Dec-18	Negative	
UPE 1317	*Rousettus aegyptiacus*	Population-level faecal	4-Dec-18	Negative	
UPE 1318	*Rousettus aegyptiacus*	Population-level faecal	4-Dec-18	Negative	
UPE 1319	*Rousettus aegyptiacus*	Population-level faecal	4-Dec-18	Negative	
UPE 1320	*Rousettus aegyptiacus*	Population-level faecal	4-Dec-18	Negative	
UPE 1321	*Rousettus aegyptiacus*	Population-level faecal	4-Dec-18	Negative	
UPE 1322	*Rousettus aegyptiacus*	Population-level faecal	4-Dec-18	Negative	
UPE 1323	*Rousettus aegyptiacus*	Population-level faecal	4-Dec-18	Negative	
UPE 1324	*Rousettus aegyptiacus*	Population-level faecal	4-Dec-18	Negative	
UPE 1325	*Rousettus aegyptiacus*	Population-level faecal	4-Dec-18	Negative	
UPE 1326	*Rousettus aegyptiacus*	Population-level faecal	4-Dec-18	Negative	
UPE 1327	*Rousettus aegyptiacus*	Population-level faecal	4-Dec-18	Negative	
UPE 1446	*Rousettus aegyptiacus*	Population-level faecal	15-Jan-19	Negative	
UPE 1447	*Rousettus aegyptiacus*	Population-level faecal	15-Jan-19	Negative	
UPE 1448	*Rousettus aegyptiacus*	Population-level faecal	15-Jan-19	Negative	
UPE 1449	*Rousettus aegyptiacus*	Population-level faecal	15-Jan-19	Negative	
UPE 1450	*Rousettus aegyptiacus*	Population-level faecal	15-Jan-19	Negative	
UPE 1451	*Rousettus aegyptiacus*	Population-level faecal	15-Jan-19	Negative	
UPE 1452	*Rousettus aegyptiacus*	Population-level faecal	15-Jan-19	Negative	
UPE 1453	*Rousettus aegyptiacus*	Population-level faecal	15-Jan-19	Negative	
UPE 1454	*Rousettus aegyptiacus*	Population-level faecal	15-Jan-19	Negative	
UPE 1455	*Rousettus aegyptiacus*	Population-level faecal	15-Jan-19	Negative	
UPE 1456	*Rousettus aegyptiacus*	Population-level faecal	15-Jan-19	Negative	
UPE 1457	*Rousettus aegyptiacus*	Population-level faecal	15-Jan-19	Negative	
UPE 1458	*Rousettus aegyptiacus*	Population-level faecal	15-Jan-19	Negative	
UPE 1459	*Rousettus aegyptiacus*	Population-level faecal	15-Jan-19	Negative	
UPE 1460	*Rousettus aegyptiacus*	Population-level faecal	15-Jan-19	Negative	
UPE 1461	*Rousettus aegyptiacus*	Population-level faecal	15-Jan-19	Negative	
UPE 1462	*Rousettus aegyptiacus*	Population-level faecal	15-Jan-19	Negative	
UPE 1463	*Rousettus aegyptiacus*	Population-level faecal	15-Jan-19	Negative	
UPE 1464	*Rousettus aegyptiacus*	Population-level faecal	15-Jan-19	Negative	
UPE 1465	*Rousettus aegyptiacus*	Population-level faecal	15-Jan-19	Negative	
UPE 1466	*Rousettus aegyptiacus*	Population-level faecal	15-Jan-19	Negative	
UPE 1467	*Rousettus aegyptiacus*	Population-level faecal	15-Jan-19	Negative	
UPE 1468	*Rousettus aegyptiacus*	Population-level faecal	15-Jan-19	Negative	
UPE 1469	*Rousettus aegyptiacus*	Population-level faecal	15-Jan-19	Negative	
UPE 1470	*Rousettus aegyptiacus*	Population-level faecal	15-Jan-19	Negative	
UPE 1471	*Rousettus aegyptiacus*	Population-level faecal	15-Jan-19	Negative	
UPE 1472	*Rousettus aegyptiacus*	Population-level faecal	15-Jan-19	Negative	
UPE 1473	*Rousettus aegyptiacus*	Population-level faecal	15-Jan-19	Negative	
UPE 1474	*Rousettus aegyptiacus*	Population-level faecal	15-Jan-19	Negative	
UPE 1475	*Rousettus aegyptiacus*	Population-level faecal	15-Jan-19	Negative	
UPE 1476	*Rousettus aegyptiacus*	Population-level faecal	15-Jan-19	Negative	
UPE 1477	*Rousettus aegyptiacus*	Population-level faecal	15-Jan-19	Negative	
UPE 1478	*Rousettus aegyptiacus*	Population-level faecal	15-Jan-19	Negative	
UPE 1479	*Rousettus aegyptiacus*	Population-level faecal	15-Jan-19	Negative	
UPE 1480	*Rousettus aegyptiacus*	Population-level faecal	15-Jan-19	Negative	
UPE 1481	*Rousettus aegyptiacus*	Population-level faecal	15-Jan-19	Negative	
UPE 1482	*Rousettus aegyptiacus*	Population-level faecal	15-Jan-19	Negative	
**UPE 1483**	***Rousettus aegyptiacus***	**Population-level faecal**	**15-Jan-19**	**Positive**	**OR365895**
UPE 1484	*Rousettus aegyptiacus*	Population-level faecal	15-Jan-19	Negative	
UPE 1485	*Rousettus aegyptiacus*	Population-level faecal	15-Jan-19	Negative	
UPE 1547	*Rousettus aegyptiacus*	Population-level faecal	12-Feb-19	Negative	
UPE 1548	*Rousettus aegyptiacus*	Population-level faecal	12-Feb-19	Negative	
UPE 1549	*Rousettus aegyptiacus*	Population-level faecal	12-Feb-19	Negative	
UPE 1550	*Rousettus aegyptiacus*	Population-level faecal	12-Feb-19	Negative	

Boldface: Population-level faecal samples which tested positive for rubulavirus RNA

Table S1 (*continued*): Egyptian rousette bat population-level faecal samples tested for rubulavirus RNA

Laboratory number	Source/Host	Sample type	Date collected	Result	Genbank Accession Number
**UPE 1551**	***Rousettus aegyptiacus***	**Population-level faecal**	**12-Feb-19**	**Positive**	**OR365896**
UPE 1552	*Rousettus aegyptiacus*	Population-level faecal	12-Feb-19	Negative	
UPE 1553	*Rousettus aegyptiacus*	Population-level faecal	12-Feb-19	Negative	
UPE 1554	*Rousettus aegyptiacus*	Population-level faecal	12-Feb-19	Negative	
UPE 1555	*Rousettus aegyptiacus*	Population-level faecal	12-Feb-19	Negative	
**UPE 1556**	***Rousettus aegyptiacus***	**Population-level faecal**	**12-Feb-19**	**Positive**	**OR365897**
UPE 1557	*Rousettus aegyptiacus*	Population-level faecal	12-Feb-19	Negative	
**UPE 1558**	***Rousettus aegyptiacus***	**Population-level faecal**	**12-Feb-19**	**Positive**	**OR365898**
UPE 1559	*Rousettus aegyptiacus*	Population-level faecal	12-Feb-19	Negative	
UPE 1560	*Rousettus aegyptiacus*	Population-level faecal	12-Feb-19	Negative	
UPE 1562	*Rousettus aegyptiacus*	Population-level faecal	12-Feb-19	Negative	
UPE 1563	*Rousettus aegyptiacus*	Population-level faecal	12-Feb-19	Negative	
UPE 1564	*Rousettus aegyptiacus*	Population-level faecal	12-Feb-19	Negative	
UPE 1565	*Rousettus aegyptiacus*	Population-level faecal	12-Feb-19	Negative	
UPE 1566	*Rousettus aegyptiacus*	Population-level faecal	12-Feb-19	Negative	
UPE 1567	*Rousettus aegyptiacus*	Population-level faecal	12-Feb-19	Negative	
**UPE 1568**	***Rousettus aegyptiacus***	**Population-level faecal**	**12-Feb-19**	**Positive**	**OR365899**
UPE 1569	*Rousettus aegyptiacus*	Population-level faecal	12-Feb-19	Negative	
**UPE 1570**	***Rousettus aegyptiacus***	**Population-level faecal**	**12-Feb-19**	**Positive**	**OR365900**
UPE 1571	*Rousettus aegyptiacus*	Population-level faecal	12-Feb-19	Negative	
UPE 1572	*Rousettus aegyptiacus*	Population-level faecal	12-Feb-19	Negative	
UPE 1574	*Rousettus aegyptiacus*	Population-level faecal	12-Feb-19	Negative	
UPE 1575	*Rousettus aegyptiacus*	Population-level faecal	12-Feb-19	Negative	
UPE 1576	*Rousettus aegyptiacus*	Population-level faecal	12-Feb-19	Negative	
UPE 1577	*Rousettus aegyptiacus*	Population-level faecal	12-Feb-19	Negative	
UPE 1578	*Rousettus aegyptiacus*	Population-level faecal	12-Feb-19	Negative	
UPE 1579	*Rousettus aegyptiacus*	Population-level faecal	12-Feb-19	Negative	
UPE 1580	*Rousettus aegyptiacus*	Population-level faecal	12-Feb-19	Negative	
UPE 1581	*Rousettus aegyptiacus*	Population-level faecal	12-Feb-19	Negative	
**UPE 1582**	***Rousettus aegyptiacus***	**Population-level faecal**	**12-Feb-19**	**Positive**	**OR365901**
UPE 1583	*Rousettus aegyptiacus*	Population-level faecal	12-Feb-19	Negative	
UPE 1584	*Rousettus aegyptiacus*	Population-level faecal	12-Feb-19	Negative	
UPE 1585	*Rousettus aegyptiacus*	Population-level faecal	12-Feb-19	Negative	
**UPE 1586**	***Rousettus aegyptiacus***	**Population-level faecal**	**12-Feb-19**	**Positive**	**OR365902**
UPE 1587	*Rousettus aegyptiacus*	Population-level faecal	12-Feb-19	Negative	
UPE 1588	*Rousettus aegyptiacus*	Population-level faecal	12-Feb-19	Negative	
UPE 1589	*Rousettus aegyptiacus*	Population-level faecal	12-Feb-19	Negative	
UPE 1590	*Rousettus aegyptiacus*	Population-level faecal	12-Feb-19	Negative	
UPE 1591	*Rousettus aegyptiacus*	Population-level faecal	12-Feb-19	Negative	
UPE 1594	*Rousettus aegyptiacus*	Population-level faecal	12-Feb-19	Negative	
UPE 1663	*Rousettus aegyptiacus*	Population-level faecal	5-Mar-19	Negative	
UPE 1664	*Rousettus aegyptiacus*	Population-level faecal	5-Mar-19	Negative	
UPE 1665	*Rousettus aegyptiacus*	Population-level faecal	5-Mar-19	Negative	
UPE 1666	*Rousettus aegyptiacus*	Population-level faecal	5-Mar-19	Negative	
**UPE 1667**	***Rousettus aegyptiacus***	**Population-level faecal**	**5-Mar-19**	**Positive**	**OR365903**
UPE 1668	*Rousettus aegyptiacus*	Population-level faecal	5-Mar-19	Negative	
**UPE 1669**	***Rousettus aegyptiacus***	**Population-level faecal**	**5-Mar-19**	**Positive**	**OR365904**
UPE 1670	*Rousettus aegyptiacus*	Population-level faecal	5-Mar-19	Negative	
UPE 1671	*Rousettus aegyptiacus*	Population-level faecal	5-Mar-19	Negative	
UPE 1672	*Rousettus aegyptiacus*	Population-level faecal	5-Mar-19	Negative	
UPE 1673	*Rousettus aegyptiacus*	Population-level faecal	5-Mar-19	Negative	
UPE 1674	*Rousettus aegyptiacus*	Population-level faecal	5-Mar-19	Negative	
UPE 1675	*Rousettus aegyptiacus*	Population-level faecal	5-Mar-19	Negative	
UPE 1676	*Rousettus aegyptiacus*	Population-level faecal	5-Mar-19	Negative	
UPE 1677	*Rousettus aegyptiacus*	Population-level faecal	5-Mar-19	Negative	
UPE 1678	*Rousettus aegyptiacus*	Population-level faecal	5-Mar-19	Negative	
UPE 1679	*Rousettus aegyptiacus*	Population-level faecal	5-Mar-19	Negative	
UPE 1680	*Rousettus aegyptiacus*	Population-level faecal	5-Mar-19	Negative	
UPE 1681	*Rousettus aegyptiacus*	Population-level faecal	5-Mar-19	Negative	

UPE 1682	*Rousettus aegyptiacus*	Population-level faecal	5-Mar-19	Negative	
UPE 1683	*Rousettus aegyptiacus*	Population-level faecal	5-Mar-19	Negative	
UPE 1684	*Rousettus aegyptiacus*	Population-level faecal	5-Mar-19	Negative	

Boldface: Population-level faecal samples which tested positive for rubulavirus RNA

Table S1 (*continued*): Egyptian rousette bat population-level faecal samples tested for rubulavirus RNA

**Laboratory number**	**Source/Host**	**Sample type**	**Date collected**	**Result**	**Genbank Accession Number**
UPE 1685	*Rousettus aegyptiacus*	Population-level faecal	5-Mar-19	Negative	
UPE 1686	*Rousettus aegyptiacus*	Population-level faecal	5-Mar-19	Negative	
UPE 1687	*Rousettus aegyptiacus*	Population-level faecal	5-Mar-19	Negative	
UPE 1688	*Rousettus aegyptiacus*	Population-level faecal	5-Mar-19	Negative	
UPE 1689	*Rousettus aegyptiacus*	Population-level faecal	5-Mar-19	Negative	
UPE 1726	*Rousettus aegyptiacus*	Population-level faecal	3-Apr-19	Negative	
UPE 1727	*Rousettus aegyptiacus*	Population-level faecal	3-Apr-19	Negative	
UPE 1728	*Rousettus aegyptiacus*	Population-level faecal	3-Apr-19	Negative	
UPE 1729	*Rousettus aegyptiacus*	Population-level faecal	3-Apr-19	Negative	
UPE 1730	*Rousettus aegyptiacus*	Population-level faecal	3-Apr-19	Negative	
UPE 1731	*Rousettus aegyptiacus*	Population-level faecal	3-Apr-19	Negative	
UPE 1732	*Rousettus aegyptiacus*	Population-level faecal	3-Apr-19	Negative	
UPE 1733	*Rousettus aegyptiacus*	Population-level faecal	3-Apr-19	Negative	
UPE 1734	*Rousettus aegyptiacus*	Population-level faecal	3-Apr-19	Negative	
UPE 1735	*Rousettus aegyptiacus*	Population-level faecal	3-Apr-19	Negative	
UPE 1736	*Rousettus aegyptiacus*	Population-level faecal	3-Apr-19	Negative	
UPE 1737	*Rousettus aegyptiacus*	Population-level faecal	3-Apr-19	Negative	
UPE 1738	*Rousettus aegyptiacus*	Population-level faecal	3-Apr-19	Negative	
UPE 1739	*Rousettus aegyptiacus*	Population-level faecal	3-Apr-19	Negative	
UPE 1740	*Rousettus aegyptiacus*	Population-level faecal	3-Apr-19	Negative	
UPE 1741	*Rousettus aegyptiacus*	Population-level faecal	3-Apr-19	Negative	
UPE 1742	*Rousettus aegyptiacus*	Population-level faecal	3-Apr-19	Negative	
UPE 1743	*Rousettus aegyptiacus*	Population-level faecal	3-Apr-19	Negative	
UPE 1744	*Rousettus aegyptiacus*	Population-level faecal	3-Apr-19	Negative	
**UPE 1745**	***Rousettus aegyptiacus***	**Population-level faecal**	**3-Apr-19**	**Positive**	**OR365905**
UPE 1746	*Rousettus aegyptiacus*	Population-level faecal	3-Apr-19	Negative	
UPE 1747	*Rousettus aegyptiacus*	Population-level faecal	3-Apr-19	Negative	
UPE 1748	*Rousettus aegyptiacus*	Population-level faecal	3-Apr-19	Negative	
**UPE 1749**	***Rousettus aegyptiacus***	**Population-level faecal**	**3-Apr-19**	**Positive**	**OR365906**
UPE 1750	*Rousettus aegyptiacus*	Population-level faecal	3-Apr-19	Negative	
UPE 1751	*Rousettus aegyptiacus*	Population-level faecal	3-Apr-19	Negative	
UPE 1752	*Rousettus aegyptiacus*	Population-level faecal	3-Apr-19	Negative	
UPE 1753	*Rousettus aegyptiacus*	Population-level faecal	3-Apr-19	Negative	
UPE 1754	*Rousettus aegyptiacus*	Population-level faecal	3-Apr-19	Negative	
UPE 1755	*Rousettus aegyptiacus*	Population-level faecal	3-Apr-19	Negative	
UPE 1756	*Rousettus aegyptiacus*	Population-level faecal	3-Apr-19	Negative	
UPE 1757	*Rousettus aegyptiacus*	Population-level faecal	3-Apr-19	Negative	
UPE 1758	*Rousettus aegyptiacus*	Population-level faecal	3-Apr-19	Negative	
UPE 1759	*Rousettus aegyptiacus*	Population-level faecal	3-Apr-19	Negative	
UPE 1760	*Rousettus aegyptiacus*	Population-level faecal	3-Apr-19	Negative	
UPE 1761	*Rousettus aegyptiacus*	Population-level faecal	3-Apr-19	Negative	
UPE 1762	*Rousettus aegyptiacus*	Population-level faecal	3-Apr-19	Negative	
UPE 1763	*Rousettus aegyptiacus*	Population-level faecal	3-Apr-19	Negative	
**UPE 1764**	***Rousettus aegyptiacus***	**Population-level faecal**	**3-Apr-19**	**Positive**	**OR365907**
UPE 1765	*Rousettus aegyptiacus*	Population-level faecal	3-Apr-19	Negative	
UPE 1766	*Rousettus aegyptiacus*	Population-level faecal	3-Apr-19	Negative	
UPE 1767	*Rousettus aegyptiacus*	Population-level faecal	3-Apr-19	Negative	
UPE 1833	*Rousettus aegyptiacus*	Population-level faecal	15-May-19	Negative	
UPE 1834	*Rousettus aegyptiacus*	Population-level faecal	15-May-19	Negative	
UPE 1835	*Rousettus aegyptiacus*	Population-level faecal	15-May-19	Negative	
UPE 1836	*Rousettus aegyptiacus*	Population-level faecal	15-May-19	Negative	
UPE 1837	*Rousettus aegyptiacus*	Population-level faecal	15-May-19	Negative	
UPE 1838	*Rousettus aegyptiacus*	Population-level faecal	15-May-19	Negative	
**UPE 1839**	***Rousettus aegyptiacus***	**Population-level faecal**	**15-May-19**	**Positive**	**OR365908**
UPE 1840	*Rousettus aegyptiacus*	Population-level faecal	15-May-19	Negative	
UPE 1841	*Rousettus aegyptiacus*	Population-level faecal	15-May-19	Negative	
UPE 1842	*Rousettus aegyptiacus*	Population-level faecal	15-May-19	Negative	
UPE 1843	*Rousettus aegyptiacus*	Population-level faecal	15-May-19	Negative	
UPE 1844	*Rousettus aegyptiacus*	Population-level faecal	15-May-19	Negative	

**UPE 1845**	***Rousettus aegyptiacus***	**Population-level faecal**	**15-May-19**	**Positive**	**OR365909**
UPE 1846	*Rousettus aegyptiacus*	Population-level faecal	15-May-19	Negative	
UPE 1847	*Rousettus aegyptiacus*	Population-level faecal	15-May-19	Negative	
UPE 1848	*Rousettus aegyptiacus*	Population-level faecal	15-May-19	Negative	

Boldface: Population-level faecal samples which tested positive for rubulavirus RNA

Table S1 (*continued*): Egyptian rousette bat population-level faecal samples tested for rubulavirus RNA

Laboratory number	Source/Host	Sample type	Date collected	Result	Genbank Accession Number
UPE 1849	*Rousettus aegyptiacus*	Population-level faecal	15-May-19	Negative	
UPE 1850	*Rousettus aegyptiacus*	Population-level faecal	15-May-19	Negative	
UPE 1851	*Rousettus aegyptiacus*	Population-level faecal	15-May-19	Negative	
UPE 1852	*Rousettus aegyptiacus*	Population-level faecal	15-May-19	Negative	
UPE 1853	*Rousettus aegyptiacus*	Population-level faecal	15-May-19	Negative	
UPE 1854	*Rousettus aegyptiacus*	Population-level faecal	15-May-19	Negative	
UPE 1855	*Rousettus aegyptiacus*	Population-level faecal	15-May-19	Negative	
UPE 1856	*Rousettus aegyptiacus*	Population-level faecal	15-May-19	Negative	
UPE 1857	*Rousettus aegyptiacus*	Population-level faecal	15-May-19	Negative	
UPE 1858	*Rousettus aegyptiacus*	Population-level faecal	15-May-19	Negative	
UPE 1860	*Rousettus aegyptiacus*	Population-level faecal	10-Jun-19	Negative	
UPE 1861	*Rousettus aegyptiacus*	Population-level faecal	10-Jun-19	Negative	
UPE 1862	*Rousettus aegyptiacus*	Population-level faecal	10-Jun-19	Negative	
UPE 1863	*Rousettus aegyptiacus*	Population-level faecal	10-Jun-19	Negative	
UPE 1864	*Rousettus aegyptiacus*	Population-level faecal	10-Jun-19	Negative	
UPE 1865	*Rousettus aegyptiacus*	Population-level faecal	10-Jun-19	Negative	
UPE 1866	*Rousettus aegyptiacus*	Population-level faecal	10-Jun-19	Negative	
UPE 1867	*Rousettus aegyptiacus*	Population-level faecal	10-Jun-19	Negative	
UPE 1868	*Rousettus aegyptiacus*	Population-level faecal	10-Jun-19	Negative	
UPE 1869	*Rousettus aegyptiacus*	Population-level faecal	10-Jun-19	Negative	
UPE 1870	*Rousettus aegyptiacus*	Population-level faecal	10-Jun-19	Negative	
UPE 1871	*Rousettus aegyptiacus*	Population-level faecal	10-Jun-19	Negative	
**UPE 1872**	***Rousettus aegyptiacus***	**Population-level faecal**	**10-Jun-19**	**Positive**	**OR365910**
UPE 1873	*Rousettus aegyptiacus*	Population-level faecal	10-Jun-19	Negative	
UPE 1874	*Rousettus aegyptiacus*	Population-level faecal	10-Jun-19	Negative	
**UPE 1875**	***Rousettus aegyptiacus***	**Population-level faecal**	**10-Jun-19**	**Positive**	**OR365911**
UPE 1876	*Rousettus aegyptiacus*	Population-level faecal	10-Jun-19	Negative	
UPE 1877	*Rousettus aegyptiacus*	Population-level faecal	10-Jun-19	Negative	
UPE 1878	*Rousettus aegyptiacus*	Population-level faecal	10-Jun-19	Negative	
UPE 1879	*Rousettus aegyptiacus*	Population-level faecal	10-Jun-19	Negative	
UPE 1880	*Rousettus aegyptiacus*	Population-level faecal	10-Jun-19	Negative	
UPE 1881	*Rousettus aegyptiacus*	Population-level faecal	10-Jun-19	Negative	
UPE 1882	*Rousettus aegyptiacus*	Population-level faecal	10-Jun-19	Negative	
UPE 1883	*Rousettus aegyptiacus*	Population-level faecal	10-Jun-19	Negative	
UPE 1884	*Rousettus aegyptiacus*	Population-level faecal	10-Jun-19	Negative	
UPE 1885	*Rousettus aegyptiacus*	Population-level faecal	10-Jun-19	Negative	
UPE 1886	*Rousettus aegyptiacus*	Population-level faecal	10-Jun-19	Negative	
UPE 1887	*Rousettus aegyptiacus*	Population-level faecal	10-Jun-19	Negative	
UPE 1888	*Rousettus aegyptiacus*	Population-level faecal	10-Jun-19	Negative	
**UPE 1889**	***Rousettus aegyptiacus***	**Population-level faecal**	**10-Jun-19**	**Positive**	**OR365912**
UPE 1976	*Rousettus aegyptiacus*	Population-level faecal	2-Jul-19	Negative	
UPE 1977	*Rousettus aegyptiacus*	Population-level faecal	2-Jul-19	Negative	
UPE 1978	*Rousettus aegyptiacus*	Population-level faecal	2-Jul-19	Negative	
UPE 1979	*Rousettus aegyptiacus*	Population-level faecal	2-Jul-19	Negative	
UPE 1980	*Rousettus aegyptiacus*	Population-level faecal	2-Jul-19	Negative	
UPE 1981	*Rousettus aegyptiacus*	Population-level faecal	2-Jul-19	Negative	
UPE 1982	*Rousettus aegyptiacus*	Population-level faecal	2-Jul-19	Negative	
UPE 1983	*Rousettus aegyptiacus*	Population-level faecal	2-Jul-19	Negative	
UPE 1984	*Rousettus aegyptiacus*	Population-level faecal	2-Jul-19	Negative	
UPE 1985	*Rousettus aegyptiacus*	Population-level faecal	2-Jul-19	Negative	
UPE 1986	*Rousettus aegyptiacus*	Population-level faecal	2-Jul-19	Negative	
UPE 1987	*Rousettus aegyptiacus*	Population-level faecal	2-Jul-19	Negative	
UPE 1988	*Rousettus aegyptiacus*	Population-level faecal	2-Jul-19	Negative	
UPE 1989	*Rousettus aegyptiacus*	Population-level faecal	2-Jul-19	Negative	
UPE 1990	*Rousettus aegyptiacus*	Population-level faecal	2-Jul-19	Negative	
UPE 1991	*Rousettus aegyptiacus*	Population-level faecal	2-Jul-19	Negative	
UPE 1992	*Rousettus aegyptiacus*	Population-level faecal	2-Jul-19	Negative	
UPE 1993	*Rousettus aegyptiacus*	Population-level faecal	2-Jul-19	Negative	

UPE 1994	*Rousettus aegyptiacus*	Population-level faecal	2-Jul-19	Negative	
UPE 1995	*Rousettus aegyptiacus*	Population-level faecal	2-Jul-19	Negative	
UPE 1996	*Rousettus aegyptiacus*	Population-level faecal	2-Jul-19	Negative	
UPE 1997	*Rousettus aegyptiacus*	Population-level faecal	2-Jul-19	Negative	
UPE 1998	*Rousettus aegyptiacus*	Population-level faecal	2-Jul-19	Negative	

Boldface: Population-level faecal samples which tested positive for rubulavirus RNA

Table S1 (*continued*): Egyptian rousette bat population-level faecal samples tested for rubulavirus RNA

Laboratory number	Source/Host	Sample type	Date collected	Result	Genbank Accession Number
UPE 1999	*Rousettus aegyptiacus*	Population-level faecal	2-Jul-19	Negative	
UPE 2000	*Rousettus aegyptiacus*	Population-level faecal	2-Jul-19	Negative	
UPE 2075	*Rousettus aegyptiacus*	Population-level faecal	13-Aug-19	Negative	
UPE 2076	*Rousettus aegyptiacus*	Population-level faecal	13-Aug-19	Negative	
UPE 2077	*Rousettus aegyptiacus*	Population-level faecal	13-Aug-19	Negative	
UPE 2078	*Rousettus aegyptiacus*	Population-level faecal	13-Aug-19	Negative	
UPE 2079	*Rousettus aegyptiacus*	Population-level faecal	13-Aug-19	Negative	
UPE 2080	*Rousettus aegyptiacus*	Population-level faecal	13-Aug-19	Negative	
UPE 2081	*Rousettus aegyptiacus*	Population-level faecal	13-Aug-19	Negative	
UPE 2082	*Rousettus aegyptiacus*	Population-level faecal	13-Aug-19	Negative	
UPE 2083	*Rousettus aegyptiacus*	Population-level faecal	13-Aug-19	Negative	
UPE 2084	*Rousettus aegyptiacus*	Population-level faecal	13-Aug-19	Negative	
UPE 2085	*Rousettus aegyptiacus*	Population-level faecal	13-Aug-19	Negative	
UPE 2086	*Rousettus aegyptiacus*	Population-level faecal	13-Aug-19	Negative	
UPE 2087	*Rousettus aegyptiacus*	Population-level faecal	13-Aug-19	Negative	
UPE 2088	*Rousettus aegyptiacus*	Population-level faecal	13-Aug-19	Negative	
UPE 2089	*Rousettus aegyptiacus*	Population-level faecal	13-Aug-19	Negative	
UPE 2090	*Rousettus aegyptiacus*	Population-level faecal	13-Aug-19	Negative	
UPE 2091	*Rousettus aegyptiacus*	Population-level faecal	13-Aug-19	Negative	
UPE 2092	*Rousettus aegyptiacus*	Population-level faecal	13-Aug-19	Negative	
UPE 2093	*Rousettus aegyptiacus*	Population-level faecal	13-Aug-19	Negative	
UPE 2094	*Rousettus aegyptiacus*	Population-level faecal	13-Aug-19	Negative	
UPE 2095	*Rousettus aegyptiacus*	Population-level faecal	13-Aug-19	Negative	
UPE 2096	*Rousettus aegyptiacus*	Population-level faecal	13-Aug-19	Negative	
UPE 2097	*Rousettus aegyptiacus*	Population-level faecal	13-Aug-19	Negative	
UPE 2098	*Rousettus aegyptiacus*	Population-level faecal	13-Aug-19	Negative	
UPE 2099	*Rousettus aegyptiacus*	Population-level faecal	13-Aug-19	Negative	
UPE 2100	*Rousettus aegyptiacus*	Population-level faecal	13-Aug-19	Negative	
UPE 2101	*Rousettus aegyptiacus*	Population-level faecal	13-Aug-19	Negative	
UPE 2102	*Rousettus aegyptiacus*	Population-level faecal	13-Aug-19	Negative	
UPE 2103	*Rousettus aegyptiacus*	Population-level faecal	13-Aug-19	Negative	
UPE 2104	*Rousettus aegyptiacus*	Population-level faecal	13-Aug-19	Negative	
UPE 2105	*Rousettus aegyptiacus*	Population-level faecal	13-Aug-19	Negative	
UPE 2106	*Rousettus aegyptiacus*	Population-level faecal	13-Aug-19	Negative	
UPE 2107	*Rousettus aegyptiacus*	Population-level faecal	13-Aug-19	Negative	
UPE 2108	*Rousettus aegyptiacus*	Population-level faecal	13-Aug-19	Negative	
UPE 2109	*Rousettus aegyptiacus*	Population-level faecal	13-Aug-19	Negative	
UPE 2110	*Rousettus aegyptiacus*	Population-level faecal	13-Aug-19	Negative	
UPE 2111	*Rousettus aegyptiacus*	Population-level faecal	13-Aug-19	Negative	
**UPE 2112**	***Rousettus aegyptiacus***	**Population-level faecal**	**13-Aug-19**	**Positive**	**OR365913**
UPE 2113	*Rousettus aegyptiacus*	Population-level faecal	13-Aug-19	Negative	
UPE 2114	*Rousettus aegyptiacus*	Population-level faecal	13-Aug-19	Negative	
**UPE 2116**	***Rousettus aegyptiacus***	**Population-level faecal**	**2-Sep-19**	**Positive**	**OR365914**
UPE 2117	*Rousettus aegyptiacus*	Population-level faecal	2-Sep-19	Negative	
UPE 2118	*Rousettus aegyptiacus*	Population-level faecal	2-Sep-19	Negative	
UPE 2119	*Rousettus aegyptiacus*	Population-level faecal	2-Sep-19	Negative	
UPE 2120	*Rousettus aegyptiacus*	Population-level faecal	2-Sep-19	Negative	
UPE 2121	*Rousettus aegyptiacus*	Population-level faecal	2-Sep-19	Negative	
UPE 2122	*Rousettus aegyptiacus*	Population-level faecal	2-Sep-19	Negative	
UPE 2123	*Rousettus aegyptiacus*	Population-level faecal	2-Sep-19	Negative	
UPE 2124	*Rousettus aegyptiacus*	Population-level faecal	2-Sep-19	Negative	
UPE 2125	*Rousettus aegyptiacus*	Population-level faecal	2-Sep-19	Negative	
UPE 2126	*Rousettus aegyptiacus*	Population-level faecal	2-Sep-19	Negative	
UPE 2127	*Rousettus aegyptiacus*	Population-level faecal	2-Sep-19	Negative	
UPE 2128	*Rousettus aegyptiacus*	Population-level faecal	2-Sep-19	Negative	
UPE 2129	*Rousettus aegyptiacus*	Population-level faecal	2-Sep-19	Negative	
UPE 2130	*Rousettus aegyptiacus*	Population-level faecal	2-Sep-19	Negative	

UPE 2131	*Rousettus aegyptiacus*	Population-level faecal	2-Sep-19	Negative	
UPE 2132	*Rousettus aegyptiacus*	Population-level faecal	2-Sep-19	Negative	
UPE 2133	*Rousettus aegyptiacus*	Population-level faecal	2-Sep-19	Negative	
UPE 2134	*Rousettus aegyptiacus*	Population-level faecal	2-Sep-19	Negative	
UPE 2135	*Rousettus aegyptiacus*	Population-level faecal	2-Sep-19	Negative	

Boldface: Population-level faecal samples which tested positive for rubulavirus RNA

Table S1 (*continued*): Egyptian rousette bat population-level faecal samples tested for rubulavirus RNA

Laboratory number	Source/Host	Sample type	Date collected	Result	Genbank Accession Number
UPE 2136	*Rousettus aegyptiacus*	Population-level faecal	2-Sep-19	Negative	
**UPE 2137**	***Rousettus aegyptiacus***	**Population-level faecal**	**2-Sep-19**	**Positive**	**OR365915**
UPE 2138	*Rousettus aegyptiacus*	Population-level faecal	2-Sep-19	Negative	
UPE 2139	*Rousettus aegyptiacus*	Population-level faecal	2-Sep-19	Negative	
UPE 2140	*Rousettus aegyptiacus*	Population-level faecal	2-Sep-19	Negative	
UPE 2141	*Rousettus aegyptiacus*	Population-level faecal	2-Sep-19	Negative	
UPE 2142	*Rousettus aegyptiacus*	Population-level faecal	2-Sep-19	Negative	
UPE 2143	*Rousettus aegyptiacus*	Population-level faecal	2-Sep-19	Negative	
UPE 2144	*Rousettus aegyptiacus*	Population-level faecal	2-Sep-19	Negative	
UPE 2145	*Rousettus aegyptiacus*	Population-level faecal	2-Sep-19	Negative	
UPE 2146	*Rousettus aegyptiacus*	Population-level faecal	2-Sep-19	Negative	
UPE 2148	*Rousettus aegyptiacus*	Population-level faecal	2-Sep-19	Negative	
UPE 2149	*Rousettus aegyptiacus*	Population-level faecal	2-Sep-19	Negative	
UPE 2150	*Rousettus aegyptiacus*	Population-level faecal	2-Sep-19	Negative	
**UPE 2151**	***Rousettus aegyptiacus***	**Population-level faecal**	**2-Sep-19**	**Positive**	**OR365916**
UPE 2152	*Rousettus aegyptiacus*	Population-level faecal	2-Sep-19	Negative	
UPE 2153	*Rousettus aegyptiacus*	Population-level faecal	2-Sep-19	Negative	
UPE 2154	*Rousettus aegyptiacus*	Population-level faecal	2-Sep-19	Negative	
UPE 2155	*Rousettus aegyptiacus*	Population-level faecal	2-Sep-19	Negative	
UPE 2156	*Rousettus aegyptiacus*	Population-level faecal	2-Sep-19	Negative	
UPE 2157	*Rousettus aegyptiacus*	Population-level faecal	2-Sep-19	Negative	
UPE 2158	*Rousettus aegyptiacus*	Population-level faecal	2-Sep-19	Negative	
UPE 2159	*Rousettus aegyptiacus*	Population-level faecal	2-Sep-19	Negative	
UPE 2160	*Rousettus aegyptiacus*	Population-level faecal	2-Sep-19	Negative	
UPE 2161	*Rousettus aegyptiacus*	Population-level faecal	2-Sep-19	Negative	
UPE 2162	*Rousettus aegyptiacus*	Population-level faecal	2-Sep-19	Negative	
UPE 2163	*Rousettus aegyptiacus*	Population-level faecal	2-Sep-19	Negative	
UPE 2164	*Rousettus aegyptiacus*	Population-level faecal	2-Sep-19	Negative	
UPE 2165	*Rousettus aegyptiacus*	Population-level faecal	2-Sep-19	Negative	
UPE 2166	*Rousettus aegyptiacus*	Population-level faecal	2-Sep-19	Negative	
UPE 2167	*Rousettus aegyptiacus*	Population-level faecal	2-Sep-19	Negative	
UPE 2168	*Rousettus aegyptiacus*	Population-level faecal	2-Sep-19	Negative	
UPE 2169	*Rousettus aegyptiacus*	Population-level faecal	2-Sep-19	Negative	
UPE 2170	*Rousettus aegyptiacus*	Population-level faecal	2-Sep-19	Negative	
UPE 2171	*Rousettus aegyptiacus*	Population-level faecal	2-Sep-19	Negative	
UPE 2172	*Rousettus aegyptiacus*	Population-level faecal	2-Sep-19	Negative	
UPE 2173	*Rousettus aegyptiacus*	Population-level faecal	2-Sep-19	Negative	
UPE 2174	*Rousettus aegyptiacus*	Population-level faecal	2-Sep-19	Negative	
UPE 2175	*Rousettus aegyptiacus*	Population-level faecal	2-Sep-19	Negative	
UPE 2300	*Rousettus aegyptiacus*	Population-level faecal	1-Oct-19	Negative	
UPE 2301	*Rousettus aegyptiacus*	Population-level faecal	1-Oct-19	Negative	
UPE 2304	*Rousettus aegyptiacus*	Population-level faecal	1-Oct-19	Negative	
UPE 2305	*Rousettus aegyptiacus*	Population-level faecal	1-Oct-19	Negative	
UPE 2306	*Rousettus aegyptiacus*	Population-level faecal	1-Oct-19	Negative	
UPE 2307	*Rousettus aegyptiacus*	Population-level faecal	1-Oct-19	Negative	
UPE 2308	*Rousettus aegyptiacus*	Population-level faecal	1-Oct-19	Negative	
UPE 2309	*Rousettus aegyptiacus*	Population-level faecal	1-Oct-19	Negative	
UPE 2310	*Rousettus aegyptiacus*	Population-level faecal	1-Oct-19	Negative	
UPE 2311	*Rousettus aegyptiacus*	Population-level faecal	1-Oct-19	Negative	
UPE 2312	*Rousettus aegyptiacus*	Population-level faecal	1-Oct-19	Negative	
UPE 2313	*Rousettus aegyptiacus*	Population-level faecal	1-Oct-19	Negative	
UPE 2314	*Rousettus aegyptiacus*	Population-level faecal	1-Oct-19	Negative	
UPE 2315	*Rousettus aegyptiacus*	Population-level faecal	1-Oct-19	Negative	
UPE 2316	*Rousettus aegyptiacus*	Population-level faecal	1-Oct-19	Negative	
UPE 2317	*Rousettus aegyptiacus*	Population-level faecal	1-Oct-19	Negative	
UPE 2318	*Rousettus aegyptiacus*	Population-level faecal	1-Oct-19	Negative	
UPE 2319	*Rousettus aegyptiacus*	Population-level faecal	1-Oct-19	Negative	

UPE 2320	*Rousettus aegyptiacus*	Population-level faecal	1-Oct-19	Negative	
UPE 2321	*Rousettus aegyptiacus*	Population-level faecal	1-Oct-19	Negative	
UPE 2322	*Rousettus aegyptiacus*	Population-level faecal	1-Oct-19	Negative	
UPE 2323	*Rousettus aegyptiacus*	Population-level faecal	1-Oct-19	Negative	
UPE 2324	*Rousettus aegyptiacus*	Population-level faecal	1-Oct-19	Negative	

Boldface: Population-level faecal samples which tested positive for rubulavirus RNA

Table S1 (*continued*): Egyptian rousette bat population-level faecal samples tested for rubulavirus RNA

Laboratory number	Source/Host	Sample type	Date collected	Result	Genbank Accession Number
UPE 2325	*Rousettus aegyptiacus*	Population-level faecal	1-Oct-19	Negative	
UPE 2326	*Rousettus aegyptiacus*	Population-level faecal	1-Oct-19	Negative	
UPE 2327	*Rousettus aegyptiacus*	Population-level faecal	1-Oct-19	Negative	
UPE 2328	*Rousettus aegyptiacus*	Population-level faecal	1-Oct-19	Negative	
UPE 2329	*Rousettus aegyptiacus*	Population-level faecal	1-Oct-19	Negative	
UPE 2330	*Rousettus aegyptiacus*	Population-level faecal	1-Oct-19	Negative	
UPE 2331	*Rousettus aegyptiacus*	Population-level faecal	1-Oct-19	Negative	
UPE 2332	*Rousettus aegyptiacus*	Population-level faecal	1-Oct-19	Negative	
UPE 2333	*Rousettus aegyptiacus*	Population-level faecal	1-Oct-19	Negative	
UPE 2334	*Rousettus aegyptiacus*	Population-level faecal	1-Oct-19	Negative	
UPE 2335	*Rousettus aegyptiacus*	Population-level faecal	1-Oct-19	Negative	
UPE 2336	*Rousettus aegyptiacus*	Population-level faecal	1-Oct-19	Negative	
UPE 2337	*Rousettus aegyptiacus*	Population-level faecal	1-Oct-19	Negative	
UPE 2338	*Rousettus aegyptiacus*	Population-level faecal	1-Oct-19	Negative	
UPE 2339	*Rousettus aegyptiacus*	Population-level faecal	1-Oct-19	Negative	
**UPE 2340**	***Rousettus aegyptiacus***	**Population-level faecal**	**1-Oct-19**	**Positive**	**OR365917**
UPE 2341	*Rousettus aegyptiacus*	Population-level faecal	1-Oct-19	Negative	
UPE 2342	*Rousettus aegyptiacus*	Population-level faecal	1-Oct-19	Negative	
UPE 2343	*Rousettus aegyptiacus*	Population-level faecal	1-Oct-19	Negative	
UPE 2344	*Rousettus aegyptiacus*	Population-level faecal	1-Oct-19	Negative	
UPE 2345	*Rousettus aegyptiacus*	Population-level faecal	1-Oct-19	Negative	
UPE 2346	*Rousettus aegyptiacus*	Population-level faecal	1-Oct-19	Negative	
UPE 2347	*Rousettus aegyptiacus*	Population-level faecal	1-Oct-19	Negative	
UPE 2348	*Rousettus aegyptiacus*	Population-level faecal	1-Oct-19	Negative	
UPE 2349	*Rousettus aegyptiacus*	Population-level faecal	1-Oct-19	Negative	
UPE 2350	*Rousettus aegyptiacus*	Population-level faecal	1-Oct-19	Negative	
UPE 2351	*Rousettus aegyptiacus*	Population-level faecal	1-Oct-19	Negative	
UPE 2352	*Rousettus aegyptiacus*	Population-level faecal	1-Oct-19	Negative	
UPE 2353	*Rousettus aegyptiacus*	Population-level faecal	1-Oct-19	Negative	
UPE 2354	*Rousettus aegyptiacus*	Population-level faecal	1-Oct-19	Negative	
UPE 2355	*Rousettus aegyptiacus*	Population-level faecal	1-Oct-19	Negative	
UPE 2356	*Rousettus aegyptiacus*	Population-level faecal	1-Oct-19	Negative	
UPE 2422	*Rousettus aegyptiacus*	Population-level faecal	6-Nov-19	Negative	
UPE 2423	*Rousettus aegyptiacus*	Population-level faecal	6-Nov-19	Negative	
UPE 2424	*Rousettus aegyptiacus*	Population-level faecal	6-Nov-19	Negative	
UPE 2425	*Rousettus aegyptiacus*	Population-level faecal	6-Nov-19	Negative	
UPE 2426	*Rousettus aegyptiacus*	Population-level faecal	6-Nov-19	Negative	
UPE 2427	*Rousettus aegyptiacus*	Population-level faecal	6-Nov-19	Negative	
**UPE 2428**	***Rousettus aegyptiacus***	**Population-level faecal**	**6-Nov-19**	**Positive**	**OR365918**
UPE 2429	*Rousettus aegyptiacus*	Population-level faecal	6-Nov-19	Negative	
UPE 2430	*Rousettus aegyptiacus*	Population-level faecal	6-Nov-19	Negative	
UPE 2431	*Rousettus aegyptiacus*	Population-level faecal	6-Nov-19	Negative	
UPE 2432	*Rousettus aegyptiacus*	Population-level faecal	6-Nov-19	Negative	
UPE 2433	*Rousettus aegyptiacus*	Population-level faecal	6-Nov-19	Negative	
UPE 2434	*Rousettus aegyptiacus*	Population-level faecal	6-Nov-19	Negative	
UPE 2435	*Rousettus aegyptiacus*	Population-level faecal	6-Nov-19	Negative	
UPE 2436	*Rousettus aegyptiacus*	Population-level faecal	6-Nov-19	Negative	
UPE 2437	*Rousettus aegyptiacus*	Population-level faecal	6-Nov-19	Negative	
UPE 2438	*Rousettus aegyptiacus*	Population-level faecal	6-Nov-19	Negative	
UPE 2439	*Rousettus aegyptiacus*	Population-level faecal	6-Nov-19	Negative	
UPE 2440	*Rousettus aegyptiacus*	Population-level faecal	6-Nov-19	Negative	
UPE 2441	*Rousettus aegyptiacus*	Population-level faecal	6-Nov-19	Negative	
UPE 2442	*Rousettus aegyptiacus*	Population-level faecal	6-Nov-19	Negative	
UPE 2443	*Rousettus aegyptiacus*	Population-level faecal	6-Nov-19	Negative	
UPE 2444	*Rousettus aegyptiacus*	Population-level faecal	6-Nov-19	Negative	
UPE 2445	*Rousettus aegyptiacus*	Population-level faecal	6-Nov-19	Negative	
**UPE 2446**	***Rousettus aegyptiacus***	**Population-level faecal**	**6-Nov-19**	**Positive**	**OR365919**

UPE 2447	*Rousettus aegyptiacus*	Population-level faecal	6-Nov-19	Negative	
UPE 2448	*Rousettus aegyptiacus*	Population-level faecal	6-Nov-19	Negative	
UPE 2449	*Rousettus aegyptiacus*	Population-level faecal	6-Nov-19	Negative	
UPE 2450	*Rousettus aegyptiacus*	Population-level faecal	6-Nov-19	Negative	
UPE 2451	*Rousettus aegyptiacus*	Population-level faecal	6-Nov-19	Negative	

Boldface: Population-level faecal samples which tested positive for rubulavirus RNA

Table S1 (*continued*): Egyptian rousette bat population-level faecal samples tested for rubulavirus RNA

**Laboratory number**	**Source/Host**	**Sample type**	**Date collected**	**Result**	**Genbank Accession Number**
UPE 2452	*Rousettus aegyptiacus*	Population-level faecal	6-Nov-19	Negative	
UPE 2453	*Rousettus aegyptiacus*	Population-level faecal	6-Nov-19	Negative	
UPE 2454	*Rousettus aegyptiacus*	Population-level faecal	6-Nov-19	Negative	
UPE 2455	*Rousettus aegyptiacus*	Population-level faecal	6-Nov-19	Negative	
UPE 2456	*Rousettus aegyptiacus*	Population-level faecal	6-Nov-19	Negative	
UPE 2457	*Rousettus aegyptiacus*	Population-level faecal	6-Nov-19	Negative	
UPE 2458	*Rousettus aegyptiacus*	Population-level faecal	6-Nov-19	Negative	
UPE 2459	*Rousettus aegyptiacus*	Population-level faecal	6-Nov-19	Negative	
UPE 2460	*Rousettus aegyptiacus*	Population-level faecal	6-Nov-19	Negative	
UPE 2461	*Rousettus aegyptiacus*	Population-level faecal	6-Nov-19	Negative	
UPE 2462	*Rousettus aegyptiacus*	Population-level faecal	6-Nov-19	Negative	
UPE 2463	*Rousettus aegyptiacus*	Population-level faecal	6-Nov-19	Negative	
UPE 2464	*Rousettus aegyptiacus*	Population-level faecal	6-Nov-19	Negative	
UPE 2465	*Rousettus aegyptiacus*	Population-level faecal	6-Nov-19	Negative	
UPE 2466	*Rousettus aegyptiacus*	Population-level faecal	6-Nov-19	Negative	
UPE 2467	*Rousettus aegyptiacus*	Population-level faecal	6-Nov-19	Negative	
UPE 2468	*Rousettus aegyptiacus*	Population-level faecal	6-Nov-19	Negative	
UPE 2469	*Rousettus aegyptiacus*	Population-level faecal	6-Nov-19	Negative	
UPE 2470	*Rousettus aegyptiacus*	Population-level faecal	6-Nov-19	Negative	
UPE 2471	*Rousettus aegyptiacus*	Population-level faecal	6-Nov-19	Negative	
UPE 2472	*Rousettus aegyptiacus*	Population-level faecal	6-Nov-19	Negative	
UPE 2473	*Rousettus aegyptiacus*	Population-level faecal	6-Nov-19	Negative	
UPE 2474	*Rousettus aegyptiacus*	Population-level faecal	6-Nov-19	Negative	
UPE 2475	*Rousettus aegyptiacus*	Population-level faecal	6-Nov-19	Negative	
UPE 2476	*Rousettus aegyptiacus*	Population-level faecal	6-Nov-19	Negative	
UPE 2477	*Rousettus aegyptiacus*	Population-level faecal	6-Nov-19	Negative	
UPE 2478	*Rousettus aegyptiacus*	Population-level faecal	6-Nov-19	Negative	
UPE 2479	*Rousettus aegyptiacus*	Population-level faecal	6-Nov-19	Negative	
UPE 2480	*Rousettus aegyptiacus*	Population-level faecal	6-Nov-19	Negative	
UPE 2481	*Rousettus aegyptiacus*	Population-level faecal	6-Nov-19	Negative	
UPE 2482	*Rousettus aegyptiacus*	Population-level faecal	6-Nov-19	Negative	
UPE 2538	*Rousettus aegyptiacus*	Population-level faecal	2-Dec-19	Negative	
UPE 2539	*Rousettus aegyptiacus*	Population-level faecal	2-Dec-19	Negative	
UPE 2540	*Rousettus aegyptiacus*	Population-level faecal	2-Dec-19	Negative	
UPE 2541	*Rousettus aegyptiacus*	Population-level faecal	2-Dec-19	Negative	
UPE 2542	*Rousettus aegyptiacus*	Population-level faecal	2-Dec-19	Negative	
UPE 2543	*Rousettus aegyptiacus*	Population-level faecal	2-Dec-19	Negative	
UPE 2544	*Rousettus aegyptiacus*	Population-level faecal	2-Dec-19	Negative	
UPE 2545	*Rousettus aegyptiacus*	Population-level faecal	2-Dec-19	Negative	
UPE 2546	*Rousettus aegyptiacus*	Population-level faecal	2-Dec-19	Negative	
UPE 2547	*Rousettus aegyptiacus*	Population-level faecal	2-Dec-19	Negative	
UPE 2548	*Rousettus aegyptiacus*	Population-level faecal	2-Dec-19	Negative	
UPE 2549	*Rousettus aegyptiacus*	Population-level faecal	2-Dec-19	Negative	
UPE 2550	*Rousettus aegyptiacus*	Population-level faecal	2-Dec-19	Negative	
UPE 2551	*Rousettus aegyptiacus*	Population-level faecal	2-Dec-19	Negative	
UPE 2552	*Rousettus aegyptiacus*	Population-level faecal	2-Dec-19	Negative	
UPE 2553	*Rousettus aegyptiacus*	Population-level faecal	2-Dec-19	Negative	
UPE 2554	*Rousettus aegyptiacus*	Population-level faecal	2-Dec-19	Negative	
UPE 2555	*Rousettus aegyptiacus*	Population-level faecal	2-Dec-19	Negative	
UPE 2556	*Rousettus aegyptiacus*	Population-level faecal	2-Dec-19	Negative	
UPE 2557	*Rousettus aegyptiacus*	Population-level faecal	2-Dec-19	Negative	
UPE 2558	*Rousettus aegyptiacus*	Population-level faecal	2-Dec-19	Negative	
UPE 2559	*Rousettus aegyptiacus*	Population-level faecal	2-Dec-19	Negative	
UPE 2560	*Rousettus aegyptiacus*	Population-level faecal	2-Dec-19	Negative	
UPE 2561	*Rousettus aegyptiacus*	Population-level faecal	2-Dec-19	Negative	
UPE 2562	*Rousettus aegyptiacus*	Population-level faecal	2-Dec-19	Negative	
UPE 2563	*Rousettus aegyptiacus*	Population-level faecal	2-Dec-19	Negative	

UPE 2564	*Rousettus aegyptiacus*	Population-level faecal	2-Dec-19	Negative	
UPE 2565	*Rousettus aegyptiacus*	Population-level faecal	2-Dec-19	Negative	
UPE 2566	*Rousettus aegyptiacus*	Population-level faecal	2-Dec-19	Negative	
UPE 2567	*Rousettus aegyptiacus*	Population-level faecal	2-Dec-19	Negative	
UPE 2568	*Rousettus aegyptiacus*	Population-level faecal	2-Dec-19	Negative	

Boldface: Population-level faecal samples which tested positive for rubulavirus RNA

Table S1 (*continued*): Egyptian rousette bat population-level faecal samples tested for rubulavirus RNA

Laboratory number	Source/Host	Sample type	Date collected	Result	Genbank Accession Number
UPE 2569	*Rousettus aegyptiacus*	Population-level faecal	2-Dec-19	Negative	
UPE 2570	*Rousettus aegyptiacus*	Population-level faecal	2-Dec-19	Negative	
UPE 2571	*Rousettus aegyptiacus*	Population-level faecal	2-Dec-19	Negative	
UPE 2572	*Rousettus aegyptiacus*	Population-level faecal	2-Dec-19	Negative	
UPE 2573	*Rousettus aegyptiacus*	Population-level faecal	2-Dec-19	Negative	
UPE 2576	*Rousettus aegyptiacus*	Population-level faecal	2-Dec-19	Negative	
UPE 2577	*Rousettus aegyptiacus*	Population-level faecal	2-Dec-19	Negative	
UPE 2578	*Rousettus aegyptiacus*	Population-level faecal	2-Dec-19	Negative	
UPE 2579	*Rousettus aegyptiacus*	Population-level faecal	2-Dec-19	Negative	
UPE 2580	*Rousettus aegyptiacus*	Population-level faecal	2-Dec-19	Negative	
UPE 2581	*Rousettus aegyptiacus*	Population-level faecal	2-Dec-19	Negative	
UPE 2582	*Rousettus aegyptiacus*	Population-level faecal	2-Dec-19	Negative	
UPE 2583	*Rousettus aegyptiacus*	Population-level faecal	2-Dec-19	Negative	
UPE 2584	*Rousettus aegyptiacus*	Population-level faecal	2-Dec-19	Negative	
**UPE 2585**	***Rousettus aegyptiacus***	**Population-level faecal**	**2-Dec-19**	**Positive**	**OR365920**
UPE 2586	*Rousettus aegyptiacus*	Population-level faecal	2-Dec-19	Negative	
UPE 2587	*Rousettus aegyptiacus*	Population-level faecal	2-Dec-19	Negative	
UPE 2588	*Rousettus aegyptiacus*	Population-level faecal	2-Dec-19	Negative	
UPE 2589	*Rousettus aegyptiacus*	Population-level faecal	2-Dec-19	Negative	
UPE 2590	*Rousettus aegyptiacus*	Population-level faecal	2-Dec-19	Negative	
UPE 2591	*Rousettus aegyptiacus*	Population-level faecal	2-Dec-19	Negative	
UPE 2592	*Rousettus aegyptiacus*	Population-level faecal	2-Dec-19	Negative	

Boldface: Population-level faecal samples which tested positive for rubulavirus RNA
