# Supplementary material for: Gastrointestinal Shedding of Rubulaviruses from Egyptian Rousette Bats: Temporal Dynamics and Spillover Implications
Source: Microorganisms. 2024 Dec 4;12(12):2505. doi: 10.3390/microorganisms12122505 (PMC11728649; doi:10.3390/microorganisms12122505)
Supplement: Supplementary file 1 [file microorganisms-12-02505-s001.zip › Rubulavirus _RScript1.pdf]

R Code – GAMM Analysis (Muvengi et al.)

## Sections

```
 # Packages and Functions
 # Data manipulation for analysis
 # Figure of raw data
 # Conduct analysis (by each of the three major viral groups)
 # Functions to obtain summaries from fitted model and plot results
 # Run summaries from the fitted model and plot

##### Packages and Functions ---------------------------------------------------------------------------------------------

## Packages
 ## Note: install any that are not already installed with install.packages("XXXX")
 ## This line will return FALSE if a package is not already installed (or an error will be
 ## encountered when trying to load one of the packages based on needed dependencies)
unlist(lapply(c("tidyverse", "magrittr", "ggplot2", "mgcv", "readxl", "lubridate"), require,
character.only = T))

## Theme for nicer plots
theme_set(theme_bw())
suppressWarnings(
  theme_update(
    axis.text.x = element_text(size = 16)
  , axis.text.y = element_text(size = 16)
  , axis.title.x = element_text(size = 16)
  , axis.title.y = element_text(size = 16)
  , legend.title = element_text(size = 12)
  , panel.grid.major = element_blank()
  , panel.grid.minor = element_blank()
  , strip.background = element_blank()
  , panel.margin = unit(0, "lines")
  , legend.key.size = unit(.55, "cm")
  , legend.key = element_rect(fill = "white")
  , panel.margin.y = unit(0.5, "lines")
  , panel.border = element_rect(colour = "black", fill = NA, size = 1)
  , strip.text.x = element_text(size = 16, colour = "black", face = "bold"))
)

## Function to scale pooled samples from:
 ## Burrows PM (1987). Improved estimation of pathogen transmission rates by
 ## group testing. Phytopathology 77 363–365.
correct_pooled_prevalence <- function(num_pos_pools, num_pools, pool_size) {
  x <- num_pos_pools
  n <- num_pools
  m <- pool_size
  a <- 0.5 * (m - 1) / m
  p_hat <- 1 - ((n - x + a) / (n + a))^(1 / m)
  p_hat
}
```

```
##### Data manipulation for analysis ---------------------------------------------------------------------------------

## load data
rubulavirus_samples <- read_excel("rubulavirus_samples.xlsx")

## Some cleaning
  ## Result to numeric
  ## Note: mutate in dplyr (should have been loaded at the top of this script)
rubulavirus_samples %<>%
  mutate(Result = plyr::mapvalues(Result, from = c("Negative", "Positive"), to = c(0, 1)) %>%
as.numeric()) %>%
  ## rename date
  rename(date = 5) %>%
  mutate(date = as.Date(date))

## Proportion positive across all groups on each sampling date
rubulavirus_samples.all <- rubulavirus_samples %>%
  group_by(date) %>%
  summarize(n_pos = sum(Result), n_samps = n(), prop_pos = n_pos / n_samps) %>%
  mutate(`Phylogenetic cluster` = "All", .after = "date") %>%
  mutate(adj_prop_pos = correct_pooled_prevalence(n_pos, n_samps, 3))

## Number of positive samples by group
rubulavirus_samples.sep <- rubulavirus_samples %>%
  group_by(date, `Phylogenetic cluster`) %>%
  summarize(n_pos = sum(Result)) %>%
  filter(!is.na(`Phylogenetic cluster`)) %>%
  ungroup()

## Expanded data frame to fill in 0s for each individual phylogenetic group on all of the days of
sampling
rubulavirus_samples.full <- expand.grid(
  date = unique(rubulavirus_samples.all$date)
, `Phylogenetic cluster` = unique(rubulavirus_samples.sep$`Phylogenetic cluster`)
) %>%
  left_join(., rubulavirus_samples.sep, by = c("date", "Phylogenetic cluster")) %>%
  mutate(n_pos = plyr::mapvalues(n_pos, from = NA, to = 0)) %>%
  left_join(., rubulavirus_samples.all %>% dplyr::select(date, n_samps), by = "date") %>%
  mutate(
    prop_pos     = n_pos / n_samps
  , adj_prop_pos = correct_pooled_prevalence(n_pos, n_samps, 3)
  )
```

```
##### Figure of raw data ----------------------------------------------------------------------------------------------------

rubulavirus_samples.full %>% rbind(., rubulavirus_samples.all) %>% {
  ggplot(., aes(date, prop_pos)) +
  geom_rect(
    fill = "grey95"
   , colour = NA
   , xmin = as.Date("2017-06-07"), xmax = as.Date("2018-01-01")
   , ymin = -Inf, ymax = Inf
   , alpha = 0.1
  ) +
  geom_rect(
    fill = "grey95"
   , colour = NA
   , xmin = as.Date("2019-01-01"), xmax = as.Date("2019-12-31")
   , ymin = -Inf, ymax = Inf
   , alpha = 0.1
  ) +
  geom_point(aes(size = n_samps), alpha = 0.7) +
  geom_line(aes(colour = `Phylogenetic cluster`)) +
  geom_line(aes(date, adj_prop_pos, colour = `Phylogenetic cluster`), linetype = "dashed") +
  facet_wrap(~`Phylogenetic cluster`, ncol = 1) +
  scale_size_continuous(name = "Number of
Samples", breaks = c(10, 20, 40, 60)) +
  xlab("Date") +
  ylab("Proportion Positive") +
  scale_x_continuous(
   breaks = as.Date(c(
    "2017-06-01", "2017-08-01", "2017-10-01", "2017-12-01"
   , "2018-02-01", "2018-04-01", "2018-06-01", "2018-08-01", "2018-10-01", "2018-12-01"
   , "2019-02-01", "2019-04-01", "2019-06-01", "2019-08-01", "2019-10-01", "2019-12-01"
   , "2020-02-01", "2020-04-01", "2020-06-01", "2020-08-01", "2020-10-01", "2020-12-01"
    )
    )
  , labels = c(
    "June 1, 2017", "Aug 1, 2017", "Oct 1, 2017", "Dec 1, 2017"
   , "Feb 1, 2018", "Apr 1, 2018", "Jun 1, 2018", "Aug 1, 2018", "Oct 1, 2018", "Dec 1, 2018"
   , "Feb 1, 2019", "Apr 1, 2019", "Jun 1, 2019", "Aug 1, 2019", "Oct 1, 2019", "Dec 1, 2019"
   , "Feb 1, 2020", "Apr 1, 2020", "Jun 1, 2020", "Aug 1, 2020", "Oct 1, 2020", "Dec 1, 2020"
  )) +
  theme(axis.text.x = element_text(size = 8, angle = 320, hjust = 0))
 }
```

```
##### Conduct analysis (by each of the three major viral groups) -----------------------------------------------

## Last bit of cleanup before analysis. Mostly to get day of year and overall day of study
rubulavirus_samples.full %<>%
  mutate(
    ## Note: yday in lubridate package (again, should have been loaded at the top of this script)
    doy     = yday(date)
    , dom     = mday(date)
    , month   = month(date)
    , year    = year(date)
    , year_m  = year - min(year)
    , day     = doy + 365 * year_m
    , month_c = month + 12 * year_m
    , .after  = "date") %>%
  dplyr::select(-year_m) %>%
  rename(viral_group = `Phylogenetic cluster`) %>%
  mutate(viral_group = as.factor(viral_group))


## Fit model
gam.model <- gam(
  prop_pos ~
    ## cc = Cyclic spline, which makes the model understand that day 1 and day 365 are next to each
other.
     ## Used to test for within-year seasonal patterns (that are repeated across years)
    s(doy, bs = "cc", k = 5, by = viral_group) +
    ## tp = thin plate spline. General smooth over whole study duration. Used to test long-term
changes in prevalence
     ## across the full study duration; i.e., would recover if there is an overall decline, while not forcing
a linear change
      ## over time
    s(day, bs = "tp", k = 5, by = viral_group)
, data    = rubulavirus_samples.full
, weights = n_samps
, family  = binomial(logit)
, method  = "REML"
  )
```

```
##### Functions to obtain summaries from fitted model and plot results -------------------------------------


## Functions to predict smoothly over time for nice figures
predict_gam      <- function(gam.fit, gam.data) {

gam.pred <- with(
  gam.data
, expand.grid(
    date        = seq(min(date), max(date), by = 1)
  , viral_group = levels(viral_group))
) %>% mutate(
    doy     = yday(date)
  , dom     = mday(date)
  , month   = month(date)
  , year    = year(date)
  , year_m  = year - min(year)
  , day     = doy + 365 * year_m
  , month_c = month + 12 * year_m
)

gam.pred.s <- gam.pred %>% dplyr::select(
  day, doy, viral_group
)

gam.pred.s <- cbind(
  gam.pred.s
, predict(
    gam.fit
  , gam.pred.s
  , se.fit = TRUE
  )
) %>% mutate(
  mid = plogis(fit)
, lwr = plogis(fit - 2*se.fit)
, upr = plogis(fit + 2*se.fit)
) %>% distinct()

gam.pred.s %<>% left_join(., gam.pred) %>% mutate(
  mid_adj = correct_pooled_prevalence(mid * 30, 30, 3)
, lwr_adj = correct_pooled_prevalence(lwr * 30, 30, 3)
, upr_adj = correct_pooled_prevalence(upr * 30, 30, 3)
)

gam.pred.s

}
```

```
plot_gam_fit       <- function(gam.pred, gam.data) {

gam.gg <- gam.pred %>%
  mutate(viral_group = plyr::mapvalues(
    viral_group
  , from = unique(viral_group)
  , to = c("Clade 1 (Mumps-Related)", "Clade 2 (Human parainfluenza 2-related)", "Clade 3
(Pararubulavirus-related)")
  )) %>% {
  ggplot(., aes(
    date, mid_adj
  , fill   = viral_group)) +
    geom_ribbon(aes(
      ymin = lwr_adj
    , ymax = upr_adj
    , colour = NA)
    , alpha = 0.15) +
    scale_color_brewer(palette = "Dark2", name = "Phylogenetic
Cluster", guide = "none") +
    scale_fill_brewer(palette = "Dark2", name = "Phylogenetic
Cluster", guide = "none") +
    geom_line(aes(colour = viral_group)) +

    geom_point(data = gam.data %>% mutate(viral_group = plyr::mapvalues(
      viral_group
      , from = unique(viral_group)
      , to = c("Clade 1 (Mumps-Related)", "Clade 2 (Human parainfluenza 2-related)", "Clade 3
(Pararubulavirus-related)")
    )), aes(y = adj_prop_pos, size = n_samps, colour = viral_group), alpha = 0.7) +

    facet_wrap(~viral_group, ncol = 1) +
    scale_size_continuous(name = "Number of
Samples", breaks = c(10, 20, 40, 60)) +
    xlab("Date") +
    ylab("Proportion Positive") +
        scale_x_continuous(
      breaks = as.Date(c(
       "2017-06-01", "2017-08-01", "2017-10-01", "2017-12-01"
     , "2018-02-01", "2018-04-01", "2018-06-01", "2018-08-01", "2018-10-01", "2018-12-01"
     , "2019-02-01", "2019-04-01", "2019-06-01", "2019-08-01", "2019-10-01", "2019-12-01"
     , "2020-02-01", "2020-04-01", "2020-06-01", "2020-08-01", "2020-10-01", "2020-12-01"))
    , labels = c(
       "June 1, 2017", "Aug 1, 2017", "Oct 1, 2017", "Dec 1, 2017"
     , "Feb 1, 2018", "Apr 1, 2018", "Jun 1, 2018", "Aug 1, 2018", "Oct 1, 2018", "Dec 1, 2018"
     , "Feb 1, 2019", "Apr 1, 2019", "Jun 1, 2019", "Aug 1, 2019", "Oct 1, 2019", "Dec 1, 2019"
     , "Feb 1, 2020", "Apr 1, 2020", "Jun 1, 2020", "Aug 1, 2020", "Oct 1, 2020", "Dec 1, 2020")) +
    theme(axis.text.x = element_text(size = 8, angle = 320, hjust = 0))
    }

gam.gg
}
```

```
plot_gam_diag      <- function(gam.fit) {

gam.plots <- plot(gam.fit, plot = F)
gam.partial <- lapply(gam.plots, get_gam_partial) %>% do.call("rbind", .)
gam.gg <- gam.partial %>% filter(x != "Gaussian quantiles") %>%
  mutate(x = as.numeric(x)) %>%
  mutate(param_type = ifelse(
    grepl("doy", param)
    , "Day of Year"
    , "Overall Day"
    )) %>%
  mutate(param = plyr::mapvalues(
    param
  , from = unique(param)
  , to   = c("Day:Pararubula", "Day:Parainfluenza", "Day:Mumps", "DoY:Pararubula",
"DoY:Parainfluenza", "DoY:Mumps")
  )) %>% filter(param_type == "Day of Year") %>% {
  ggplot(., aes(x, mid, colour = param, fill = param)) +
    geom_ribbon(aes(ymin = lwr, ymax = upr), alpha = 0.2) +
    geom_line() +
      scale_colour_brewer(palette = "Dark2", name = "Virus group") +
      scale_fill_brewer(palette = "Dark2", name = "Virus group") +
            xlab("Day") +
            ylab("Partial Effect (logit scale)") +
      facet_wrap(~param_type, scales = "free") +
      theme(axis.text.x = element_text(size = 13)) +
      scale_x_continuous(
        breaks = c(32, 91, 152, 213, 274, 335)
      , labels = c("Feb", "Apr", "Jun", "Aug", "Oct", "Dec")
      )}
gam.gg
}

get_gam_partial    <- function(x) {

param_name <- as.character(x$ylab)

gam.partial <- data.frame(
   x   = x$x
 , fit = x$fit
 , se  = x$se
) %>% mutate(
  lwr   = fit - 2 * se
, mid   = fit
, upr   = fit + 2 * se
, lwr_p = plogis(lwr)
, mid_p = plogis(fit)
, upr_p = plogis(upr)
) %>% mutate(param = param_name)

}
```

```
##### Run summaries from the fitted model and plot ------------------------------------------------------------

## Print summary and plot
summary(gam.model)
gam.pred <- predict_gam(gam.model, rubulavirus_samples.full)
gam.plot <- plot_gam_fit(gam.pred, rubulavirus_samples.full); gam.plot
plot_gam_diag(gam.model)
```
